# Identification of target genes of *Astragalus mongholicus* and *Saposhnikovia divaricata* extracts in human synoviocytes for potential osteoarthritis treatment

**DOI:** 10.1186/s41065-025-00581-7

**Published:** 2025-10-08

**Authors:** Jiarui Zhou, Xiaopei Gao, Xing Liu, Sitong Yang, Zhengren Wei, Yubao Gong

**Affiliations:** 1 Department of Orthopedics, the First Hospital of Jilin University, No. 1 Xinmin Street, Changchun, 130021 China; 2 School of Acupuncture and Massage College, Changchun University of Chinese Medicine, 1035 Bo Shuo Road, Changchun, 130117 China; 3 Department of Pharmacology, Jilin University College of Basic Medical Sciences, 126 Xinmin Street, Changchun, 130021 China

**Keywords:** Osteoarthritis, Synoviocytes, A. mongholicus, S. divaricata, Matrix metalloproteinase, CITED2

## Abstract

Osteoarthritis (OA) is a leading cause of pain and disability worldwide. Despite extensive research, no curative treatment is currently available. Most therapies focus on symptom relief or disease modification. Traditional Chinese medicines, particularly *Astragalus mongholicus* (Huangqi) and *Saposhnikovia divaricata* (Fangfeng), have demonstrated therapeutic potential for OA, but their molecular mechanisms remain unclear. The synovium plays a key role in OA pathogenesis and disease progression, yet the effects of these herbal extracts on synoviocytes are poorly understood. To explore potential targets, we analyzed all publicly available microarray datasets comparing gene expression in OA and healthy synovium/synoviocytes. Differentially expressed genes (DEGs) were identified and compared with known targets of *A. mongholicus* and *S. divaricata*. The intersecting genes were subjected to Gene Ontology, KEGG pathway, and protein‒protein interaction analyses to identify hub genes. Key transcription factors were predicted using the KNOCK.TF database. Expression of hub genes and transcription factors was validated in human synoviocytes treated with *A. mongholicus* and *S. divaricata* extracts. We identified ten hub genes, eight of which may serve as OA biomarkers. Three hub transcription factors—AR, CITED2, and SF1—were upregulated by the extracts, while MMP2 and MMP9, two OA-associated genes, were downregulated. Our findings suggest that *A. mongholicus* and *S. divaricata* may exert therapeutic effects on OA by modulating synoviocyte gene expression, notably through upregulation of AR and CITED2 and downregulation of MMP2 and MMP9. These herbs may provide molecular targets for novel OA treatments.

**Supplementary Information:**

The online version contains supplementary material available at 10.1186/s41065-025-00581-7.

## Introduction

Osteoarthritis (OA) is the most common form of arthritis and one of the major causes of pain and disability [1]. OA affects 7% of the global population, with more than 500 million people affected worldwide [2]. The hallmark of osteoarthritis is articular cartilage degeneration; however, OA is increasingly recognized as a disease of the whole joint [1]. In addition to articular cartilage degeneration, other major pathological changes include the formation of osteophytes, subchondral bone sclerosis, synovial inflammation, the degeneration of ligaments and the meniscus in the knee joint, and alterations in the joint capsule [1].

Two types of cells predominantly reside in the healthy synovium: type A macrophage-like cells and type B fibroblast-like synovial cells [3]. Type A synoviocytes are considered tissue-resident macrophages. They can phagocytose cell debris and waste in the joint cavity and possess an antigen-presenting ability [4]. Type B synoviocytes are the cells that have secretory functions. They secrete collagens, fibronectin [5, 6], hyaluronic acid [7], and other glycosaminoglycans into the joint cavity. Recently, accumulating knowledge of type B synoviocytes has revealed that they can dictate a pro- or anti-inflammatory state, depending on which cytokines or growth factors are present in the surrounding synovial environment [8]. Type B synoviocytes express Toll-like receptors such as TLR-2, TLR-3, and TLR-4; upon stimulation, TLRs can activate innate immune pathways and the secretion of inflammatory cytokines and metalloproteinases [9–12], which can cause inflammation in chondrocytes and cartilage degradation. Under certain circumstances, type B synoviocytes can also secrete chemoattractants, such as CCL-2, CCL-5, CCL-8, CXCL-5, and CXCL-10. These chemoattractants attract monocytes and promote macrophage infiltration [13], which can further cause the inflammation and degradation of cartilage. Therefore, activated type B synoviocytes secrete inflammatory cytokines, enzymes, and chemoattractants that exacerbate joint inflammation-induced cartilage degradation. These findings suggest that synoviocytes play very important roles in inflammation and OA pathogenesis [14]. Synovitis results from synovial inflammation, which often occurs in the early stages of OA [15].

Although the synovium plays significant roles in OA pathogenesis and disease progression [16], the synovium or synoviocytes have not received as much attention as chondrocytes have. However, recent research on the synovium or synoviocytes has enabled researchers to elucidate the mechanisms of OA pathogenesis and identify therapeutic targets in synoviocytes for OA. Based on these findings, the proposed targets mainly include proinflammatory cytokines, such as IL-1, IL-6, and TNFα; MMPs, such as MMP-2 and MMP-9; and TLRs, such as TLR-2, TLR-3 and TLR-4 [17, 18]. However, targeting catabolic cytokines and MMPs in the joint cavity has shown only limited effects [19]. Identifying more efficient therapeutic targets warrants more research. Treating OA with traditional Chinese medicine has shown a relatively better safety profile than conventional therapy, such as non-steroidal anti-inflammatory drugs [20]. We combined bioinformatics and database search techniques to identify putative targets of *A. mongholicus* and *S. divaricata* in the synovium or synoviocytes at the molecular level. This approach could provide novel therapeutic targets for OA.

No cure is available for OA thus far, and the available treatments focus on OA symptom relief or disease modification. Traditional Chinese medicine, such as Huangqi (*Astragalus mongholicus* Bunge, Fabaceae) and Fangfeng (*Saposhnikovia divaricata* (Turcz.) Schischk, Apiaceae) has shown efficacy in relieving OA symptoms [21]. However, due to the very complicated components of Chinese herbal medicine, the underlying mechanisms of *A. mongholicus* and *S. divaricata* in OA symptom relief are not known, and no studies have elucidated the mechanisms involved. With the advancement of technology, many compounds in traditional Chinese medicine have been identified. Publicly available databases such as the Traditional Chinese Medicines Integrated Database (TCMID) (http://www.megabionet.org/tcmid/, *last accessed in June 2023*) and Traditional Chinese Medicine Systems Pharmacology (TCMSP) (http://tcmspw.com/tcmsp.php, last accessed in June 2023) can be used to search for compounds in certain herbs, such as *A. mongholicus* and *S. divaricata*. *A. mongholicus* and *S. divaricata* are usually used together in traditional Chinese medicine because they have complementary properties that enhance the other’s therapeutic effects or exert synergistic effects [22]. *A. mongholicus* is known for its immune-boosting and anti-inflammatory properties, and *S. divaricata* has expectorant and anti-inflammatory effects [22]. OA is considered an inflammatory disease; therefore, *A. mongholicus* and *S. divaricata* are good candidates for potential OA symptom relief or even a cure. *A. mongholicus* and *S. divaricata* are usually used at a 1:1 ratio in traditional Chinese medicine. Some comparison studies have also indicated that a 1:1 ratio is better than other ratios [23, 24]. Based on the traditional Chinese medicine practices and the literature, we used a 1:1 ratio of *A. mongholicus* to *S. divaricata* in this study.

In this study, we utilized microarray datasets derived from OA and control synovium to identify the genes that play critical roles in OA and to subsequently identify the key transcription factors that regulate these genes. Furthermore, based on the identified hub genes and key transcription factors, we searched Chinese medicine databases for possible herbs that can target these hub genes and/or transcription factors. To our knowledge, this study is the first to systematically reveal that extracts of the Chinese medicines *A. mongholicus* and *S. divaricata* can target genes and/or transcription factors in synoviocytes, which may play important roles in OA.

## Materials and methods

### Gene expression profile datasets

We searched all publicly available datasets to identify genes that are differentially expressed between OA synoviocytes and normal synoviocytes. All datasets available for the OA gene expression analysis were downloaded from the Gene Expression Omnibus website (https://www.ncbi.nlm.nih.gov/geo). Datasets without patient characterization information were not used. As a result, four datasets, namely, GSE12021, GSE55235, GSE55457, and GSE82107, were used in the analysis. The GSE12021 dataset was obtained from 22 OA patients and 4 non-OA controls via the GPL96 Affymetrix Human Genome U133A Array (HG-U133A) platform [25]. The GSE55235 and GSE55457 datasets included 20 OA patients and 10 healthy controls and 23 OA patients and 10 healthy controls, respectively. Microarray assays were performed on the GPL96 Affymetrix Human Genome U133A Array (HG-U133 A/B) platform [26]. The GSE82107 dataset was obtained from 10 end-stage OA synovial biopsies and 7 synovial biopsies from individuals without a joint disease [27].

### Identification of differentially expressed genes (DEGs)

We utilized the Limma package in R for the differential expression analysis [28] and applied multiple comparison correction to the p values to identify the DEGs. The screening criterion was set at a p value < 0.05 after false discovery rate (FDR) adjustment. A log fold change |logFC|>1 and adjusted *p* < 0.05 were used as the criteria for DEGs. The identified DEGs were presented in a volcano plot using ggplot2 and a heatmap using Pheatmap.

### Gene Ontology (GO) and kyoto encyclopedia of genes and genomes (KEGG) enrichment assays

The identified DEGs or target genes were analyzed for enriched Gene Ontology (GO) terms using the Metascape online platform (https://metascape.org) [29], which applies a multiple comparison correction based on the Benjamin‒Hochberg method by default. Significant pathways are reported with the results adjusted through the FDR correction. The analysis focused on the biological process (BP) category. The parameters were set as follows: *p* < 0.01, minimum count > 3, and enrichment factor > 1.5. Gene set enrichment analysis (GSEA) was performed to identify the signaling pathways associated with the enriched DEGs [30]. Gene set permutations were performed 10,000 times for each analysis. Curated gene sets from the Kyoto Encyclopedia of Genes and Genomes (KEGG) pathways were used as references [31]. Adjusted *p* < 0.05 and FDR < 0.25 were set as the criteria for statistical significance. The adjusted p value and the normalized enrichment score (NES) were used to measure gene set enrichment.

### Identification of OA-associated genes targeted by A. mongholicus and S. divaricata

We first identified the genes associated with OA from two databases, i.e., GeneCards and Online Mendelian Inheritance in Man (OMIM). The GeneCards database (https://www.genecards.org/) provides comprehensive information on all annotated and predicted human genes, including genomic, transcriptomic, proteomic, clinical, genetic, and functional information [32]. Online Mendelian Inheritance in Man (OMIM) (https://omim.org/) is another comprehensive and constantly updated database of human genes and genetic disorders [33]. We used “osteoarthritis” as the keyword to retrieve the OA targets from both databases.

The chemical compounds of *A. mongholicus* and *S. divaricata* were searched in the Traditional Chinese Medicines Integrated Database (TCMID) (http://www.megabionet.org/tcmid/) and Traditional Chinese Medicine Systems Pharmacology (TCMSP) database (http://tcmspw.com/tcmsp.php). The 3D structures of the obtained compounds were subsequently searched in PubChem (https://pubchem.ncbi.nlm.nih.gov) [34]. Because structural information is necessary to predict the targets of compounds, we excluded compounds for which structural information was not available. The potential target genes of each compound were searched on PharmMapper (http://www.lilab-ecust.cn/pharmmapper/) [35]. Briefly, the following parameters were set when searching for targets in PharmMapper to identify the target genes corresponding to a compound identified in PubChem: generate conforms was set to “Yes”, maximum generate conformers was set at 300, the target dataset was selected as “Human Protein Targets Only (v2010,2241)”, and the reversed number of targets was set at 300. The following parameters were set to explore the transcription factors among the 10 hub genes using the subnetwork analysis feature of KnockTF: the number of edges was set at 100, and the species was selected as *Homo sapiens*.

### Protein‒protein interaction (PPI) network analysis

The STRING database (http://string-db.org) was used for the analysis (score > 0.4) to predict the potential interactions among the proteins encoded by the DEGs identified in the previous analysis [36]. We further used Cytoscape software to visualize the protein–protein interaction networks [37]. The STRING database employs a significance scoring mechanism to evaluate interactions. We further increased the robustness of our network analysis results by integrating multiple scoring strategies, such as maximum clique centrality (MCC), within Cytoscape. The hub genes [38]within the PPI network were identified using CytoHubba.

### Hub gene identification and predicted diagnosis

Overlapping differentially expressed genes (DEGs) were analyzed using Spearman’s correlation analysis, and hub genes were identified for further analysis. We identified eight hub genes. These genes were then used for the receiver operating characteristic (ROC) curve analysis with the pROC package [39]. The areas under the curves are presented as the results of the analysis.

### Identification of transcription factors that target hub genes

Transcription factors that target hub genes were searched in the publicly available KNOCK.TF database (*/KnockTF (licpathway.net)*).

### A. mongholicus and S. divaricata extracts

*A. mongholicus* and *S. divaricata* extracts were prepared by a traditional Chinese medicine method and a 1:1 ratio of *A. mongholicus*/*S. divaricata* was used, according to the literature [23, 24]. Briefly, 100 g of *A. mongholicus* and 100 g of *S. divaricata* were boiled in 1 L of distilled water for 1.5 h. The supernatant was removed from *A. mongholicus* and *S. divaricate* remains. Another 1 L of water was added to *A. mongholicus* and *S. divaricata*, and the mixture was boiled for another hour. The supernatant was combined with the first extract. The combined extracts from *A. mongholicus* and *S. divaricata* were then filtered and lyophilized. The lyophilized extract was stored at 4 °C until use. A total of 1 mL aliquots of three batches of the active compounds extracted from *A. mongholicus* and *S. divaricata* were analyzed by HPLC-DAD on a Thermo Vanquish HPLC system to ensure the repeatability and reliability of the *A. mongholicus* and *S. divaricata* extracts. A Welch Xtimate C8 (4.6 × 250 mm × 5 μm) column was used in the experiments. Twenty microliters of each sample was manually injected. The chromatographic experiments were performed at a 1 mL/min flow rate at room temperature, and a diode array detector (DAD) was used to monitor the UV absorption at 254 nm.

### Cell culture and total RNA isolation

The C28/I2 human chondrocyte line was purchased from iCell Biosciences (iCell Biosciences Shanghai, China) and maintained in our laboratory. The cells were cultured in Dulbecco’s modified Eagle’s medium (DMEM/F12, Thermo Fisher Scientific) supplemented with 10% FBS (Thermo Fisher Scientific). Human fibroblast-like synoviocytes and MH7A and HFLS-RA cells were obtained from QuiCell Biotechnology (Shanghai, China). MH7A and HFLS-RA cells were cultured in RPMI-1640 medium (Thermo Fisher Scientific) supplemented with 10% FBS [40]. All the cell lines were cultured at 37 °C in a humidified incubator with 5% CO_2_. The cells were serum-starved with media containing 1% FBS for 16 h and then stimulated with different concentrations of *A. mongholicus* and *S. divaricata* extracts for 12–24 h. The cells were then directly lysed with 1 mL of TRIzol (Thermo Fisher Scientific) for total RNA isolation after removing the cell culture media. The cell lysate was then processed with a Qiagen RNeasy Kit (Qiagen) according to the manufacturer’s protocol.

### Real-time PCR

In this study, we first prioritized to validate the mRNA and protein level expression hub transcription factors, because hub transcription factors represent key regulatory nodes within the OA-associated gene network. Their centrality suggests they may orchestrate broader transcriptional changes in synoviocytes, making them high-value targets for mechanistic validation. We also chose MMP2 and MMP9 mRNA and protein level expression validation, because they are well-established mediators of extracellular matrix degradation and synovial inflammation in OA. Their expression levels are directly linked to cartilage breakdown and joint damage. Total RNA extracted from treated and untreated C28/I2, HFLS-RA, and MH7A cells was used to synthesize first-strand cDNA with a ReverAid First Strand cDNA Synthesis Kit (Thermo Fisher Scientific) according to the manufacturer’s protocol. One microgram of total RNA was used in each reaction. The first-strand cDNA samples were then used as templates for real-time PCR to quantify the relative expression of mRNAs with SYBR Green (Thermo Fisher Scientific Cat# 4344463) and gene-specific primers (listed in Supplemental Table 1). The first-strand cDNA was diluted 50 times, and 5 µL was added to each reaction. The reactions were set up according to the SYBR Green master mix protocol. Real-time PCR was performed on a Bio-Rad CFX96 thermocycler using the following program: 95.0 °C for 10 min, 95.0 °C for 15 s and 60.0 °C for 1 min for 40 cycles, followed by an incubation at 4 °C. The real-time PCR data were analyzed using Bio-Rad CFX Maestro software. The gene expression level was normalized to that of the housekeeping gene (GAPDH) using the 2^−ΔΔCt^ method, as previously reported [41, 42].

### Western blot analysis

Treated and untreated cells were washed with ice-cold PBS, lysed directly in a 6-well plate with RIPA buffer and transferred to 1.5 mL centrifuge tubes after a cell scraper was used to remove all the cells from the bottom of the wells. The cell lysates were then centrifuged at 12,000 × *g* and 4 °C for 5 min to remove the debris. The proteins in the supernatant were transferred to new tubes and kept on ice. Protein concentrations were determined using Pierce BCA Protein Assay Reagent A (Thermo Fisher Scientific). Approximately 25 µg of total protein was loaded into each well, separated by sodium dodecyl sulfate‒polyacrylamide gel (SDS‒PAGE) electrophoresis on a 10% polyacrylamide gel and transferred to immunoblotting nitrocellulose (NC) membranes (Millipore). The membranes were incubated with primary antibodies against CITED2 (1:1000, ABclonal, Cat# A12831), SF1 (1:1000, Cat# ER65012), AR (1:1000, Cat# HA721156), MMP2 (1:1000, ABclonal, Cat# A6247), MMP9 (1:1000, ABclonal, Cat# A0289), or GAPDH (Proteintech Cat# 60004-1-1G), followed by three wash steps and an incubation with horseradish peroxidase-conjugated secondary antibodies (Beyotime, Beijing, China). The binding of primary and secondary antibodies was visualized using the ECL Western blotting analysis system after excessive unbound secondary antibodies was washed three times. The protein marker used for evaluating the molecular weights of the target proteins was purchased from Servicebio (Cat# G2086).

### Statistical analysis

Statistical analyses were performed using Excel or GraphPad Prism software. All the data described in this manuscript are presented as the means ± standard deviations. A P value < 0.05 was considered to indicate statistical significance.

## Results

### Target genes of the active compounds from A. mongholicus and S. divaricata

A total of 260 compounds from *A. mongholicus* and *S. divaricata* were obtained from the TCMID and TCMSP database searches. Based on their oral bioavailability and drug-likeness, we identified 20 potentially active compounds from *A. mongholicus* and 18 candidate active compounds from *S. divaricata*. These 38 compounds were further screened for 3D structure availability. Finally, we identified 28 compounds (17 from *A. mongholicus* and 11 from *S. divaricata*) as potential active compounds. The characteristics of these compounds are listed in Supplemental Table 2. Using PharmMapper, we searched for potential target genes of each compound, and the top 30 genes were selected for further analysis. A total of 281 genes were identified as potential target genes of the active compounds. We also identified 2612 genes associated with OA from the GeneCards and OMIM databases. The overlapping OA-associated genes and potential target genes of the active compounds (58 genes, Supplemental Table 3) were further analyzed.

### DEG identification

**Fig. 1:**
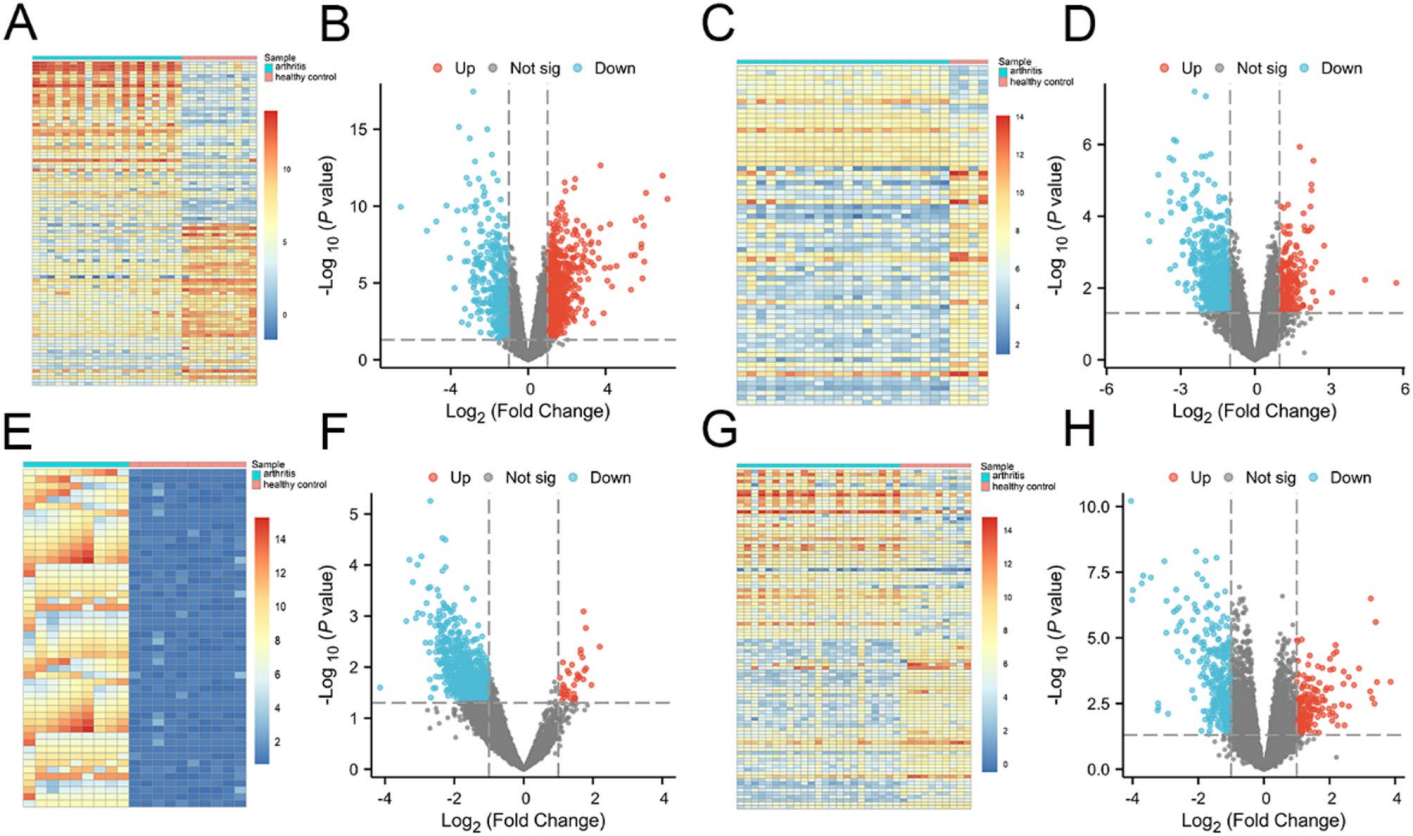
DEGs were identified in each dataset. **A** and **B**, DEGs from dataset GSE55235. **C** and **D**, DEGs from dataset GSE12021. **E** and **F**, DEGs from dataset GSE182107. G and H, DEGs from dataset GSE55457

Because each dataset derived from the GEO website was obtained through different experiments, cross-dataset comparisons may increase the number of false results. Therefore, we analyzed each dataset individually to obtain the differentially expressed genes (DEGs). Candidate DEGs were screened using the Limma package and the criteria of a log fold change |logFC|>1 and *P* < 0.05 [28]. The upregulated and downregulated DEGs between OA samples and controls from each dataset were identified and visualized in heatmaps (Fig. 1A, C, E, G) and volcano plots (Fig. 1B, D, F, H). The overlapping DEGs were further identified from the DEGs of these 4 datasets. As a result, we identified a total of 258 DEGs that overlapped in these 4 datasets (Supplemental Fig. 1A). We further identified 46 overlapping genes between the 258 DEGs and the 58 target genes of the traditional Chinese medicine *A. mongholicus* and *S. divaricata* (Supplemental Fig. 1B, and Supplemental Table 4).

**Fig. 2:**
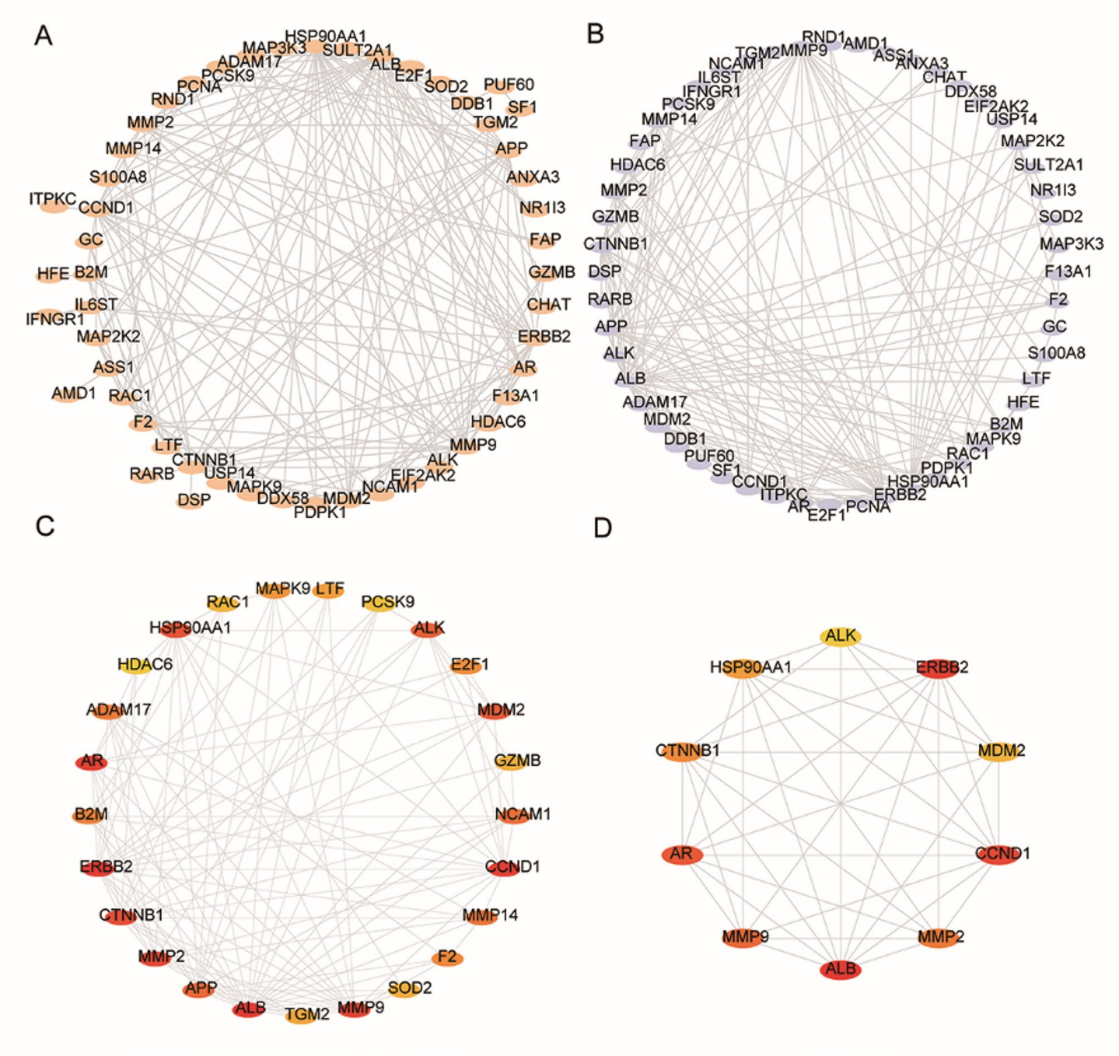
Ten hub genes were identified through PPI network construction. **A** and **B**, protein protein interaction network construction based on the overlapped DEGs. **C** and **D**, identification of hub genes

### Protein‒protein interaction (PPI) network analysis and hub gene identification

**Fig. 3:**
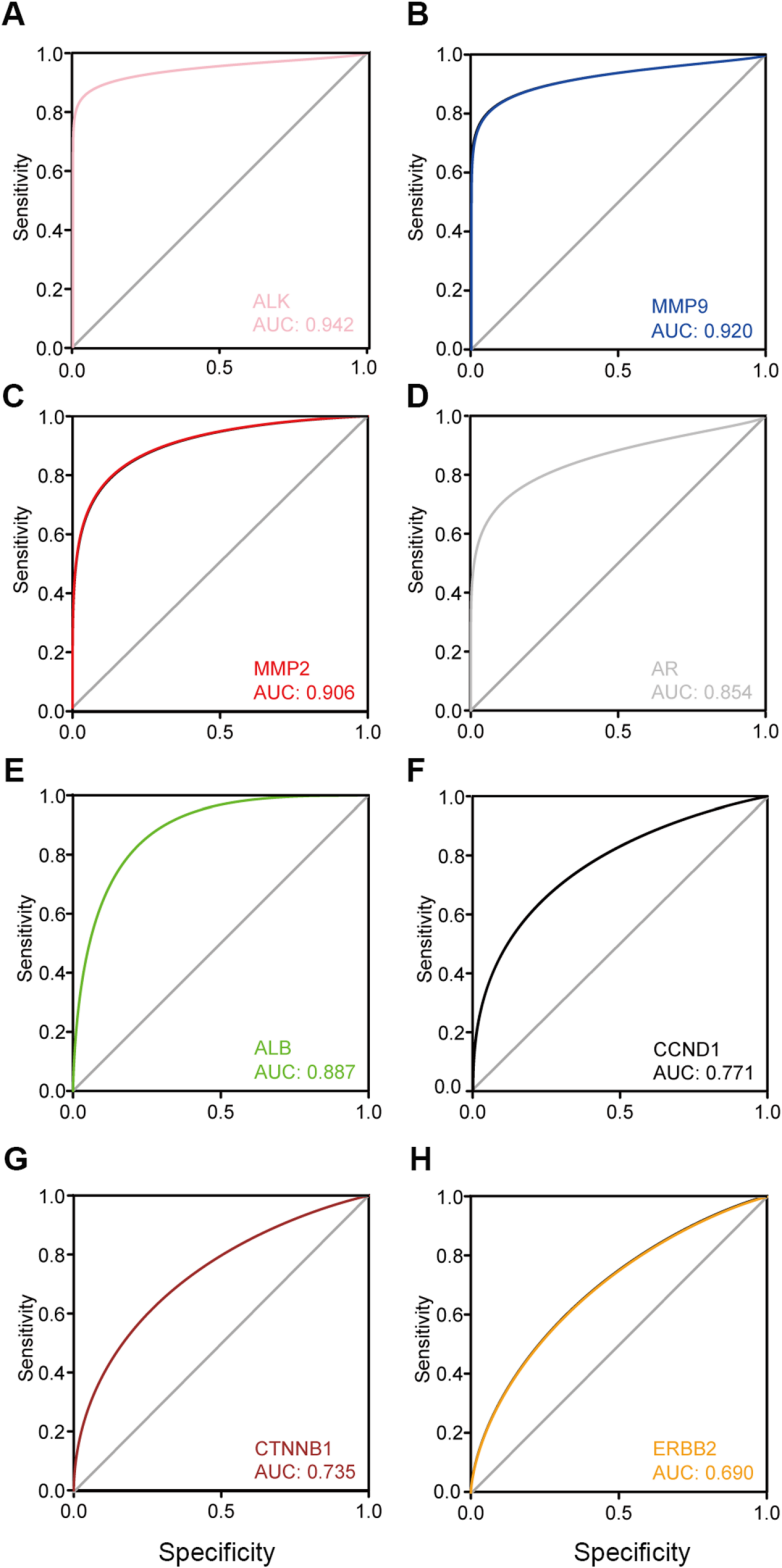
Hub genes can potentially be used as OA biomarkers. **A-H**, ROC analysis of each hub gene showed the area under curve (AUC)

We aimed to further understand the potential interactions between the proteins encoded by the 46 DEGs, and we identified that they potentially play roles in osteoarthritis and can also be targeted by the traditional Chinese medicine *(A) mongholicus* and *S. divaricata*. We performed a protein–protein interaction (PPI) analysis. Two subnetworks were identified, as shown in Fig. 2A, (B) We further identified the top 10 hub genes, as shown in Fig. 2C, D. Some of the proteins encoded by these hub genes, such as MMP9, MMP2 [43], Cyclin D1 (CCND1) [44, 45], and β-catenin [29, 46–48], are known to play important roles in osteoarthritis. No experimental evidence is available that other proteins, such as anaplastic lymphoma receptor tyrosine kinase (ALK) and Erb-B2 receptor tyrosine kinase 2 (ERBB2), play roles in osteoarthritis. Therefore, the identification of these hub genes may lead to new avenues for osteoarthritis treatment. We further elucidated whether the hub genes can be used as biomarkers to predict OA by analyzing receiver operating characteristic (ROC) curves for the GSE55235 dataset. The areas under the curve for ALK, MMP9, and MMP2 were high (AUC > 0.9, Fig. 3A-C). The other markers had AUCs of 0.69–0.887 (Fig. 3E‒H). MMP9 and MMP2 expression in chondrocytes has been correlated with OA [43]. Interestingly, ALK and the androgen receptor (AR) also had very good AUCs (0.942 and 0.854, respectively; Fig. 3A, D). Although the hub genes need to be further validated in validation dataset(s), these data indicate that the identified hub genes could very likely serve as osteoarthritis biomarkers in synoviocytes.

### Identification of hub transcription factors and their target genes

Upstream common transcription factors (or cofactors) that regulate the final 46 overlapping DEGs may be used as drug targets for OA symptom relief or even a cure. Therefore, we constructed transcription factor (or co-factor)–target gene interaction networks. We identified three key hub transcription factors (or cofactors): androgen receptor (AR), Cbp/P300 interacting transactivator with Glu/Asp-rich carboxy-terminal domain 2 (CITED2), and splicing factor 1 (SF1). CITED2 has been proven to play a role in OA in chondrocytes [49, 50], but whether CITED2 plays any role in OA in synoviocytes has not been elucidated. Whether AR and SF1 play any roles in OA is not clear, which indicates that our findings may lead to a new direction of OA research.

**Fig. 4:**
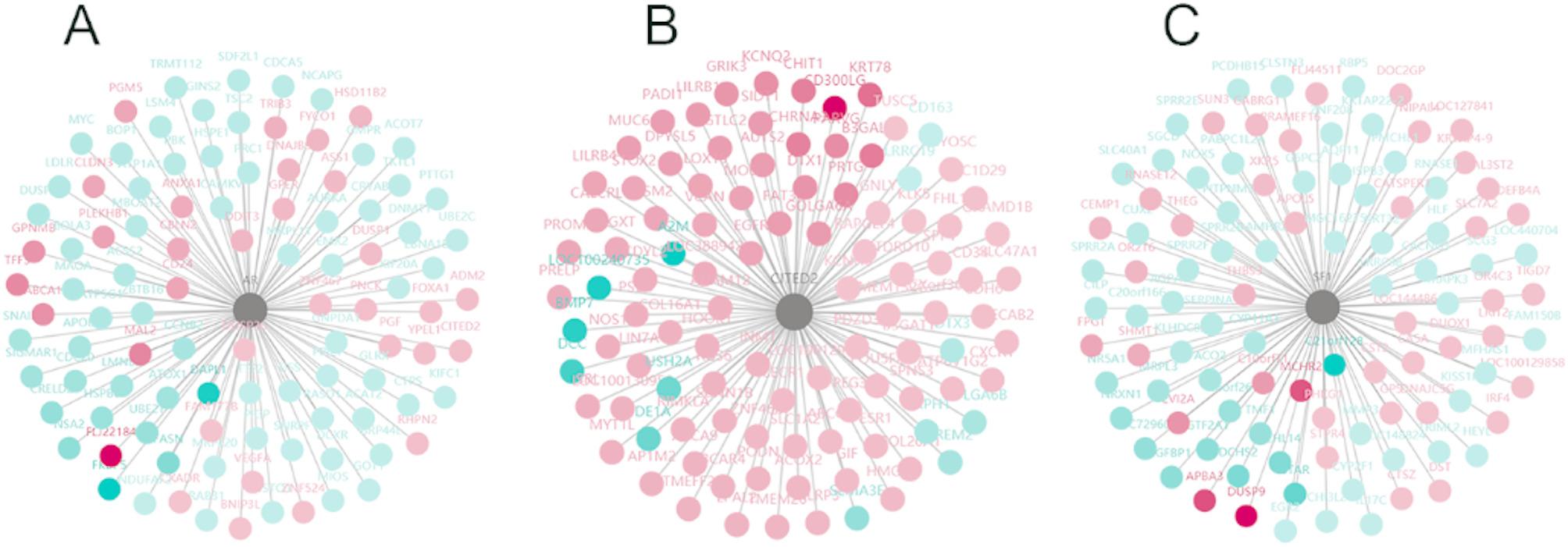
Three hub transcription factors were identified based on the DEGs. **A**, hub transcription factor AR target gene network. **B**, hub transcription factor CITED2 target gene network. **C**, hub transcription factor SF-1 target gene network

We further explored the target genes of these identified hub transcription (co-)factors. Figure 4A shows the interaction network of AR target genes. KEGG and Gene Ontology analyses revealed that the functions of the AR target genes involved cell cycle regulation (Supplemental Fig. 2A, B). CITED2 target genes were mainly involved in hormone regulation (Fig. 4D, Supplemental Fig. 2 C) and the NOTCH signaling pathway (Supplemental Fig. 2 D). Interestingly, SF1 target genes were enriched in the negative regulation of cytokine secretion, the NFκB signaling pathway, and the TGFβ signaling pathway (Fig. 4G, Supplemental Fig. 2 E, F). These pathways further indicate that these hub transcription factors may play significant roles in OA in synoviocytes.

### A. mongholicus and S. divaricata extracts stimulated the expression of three hub transcription factors in synoviocytes

**Fig. 5:**
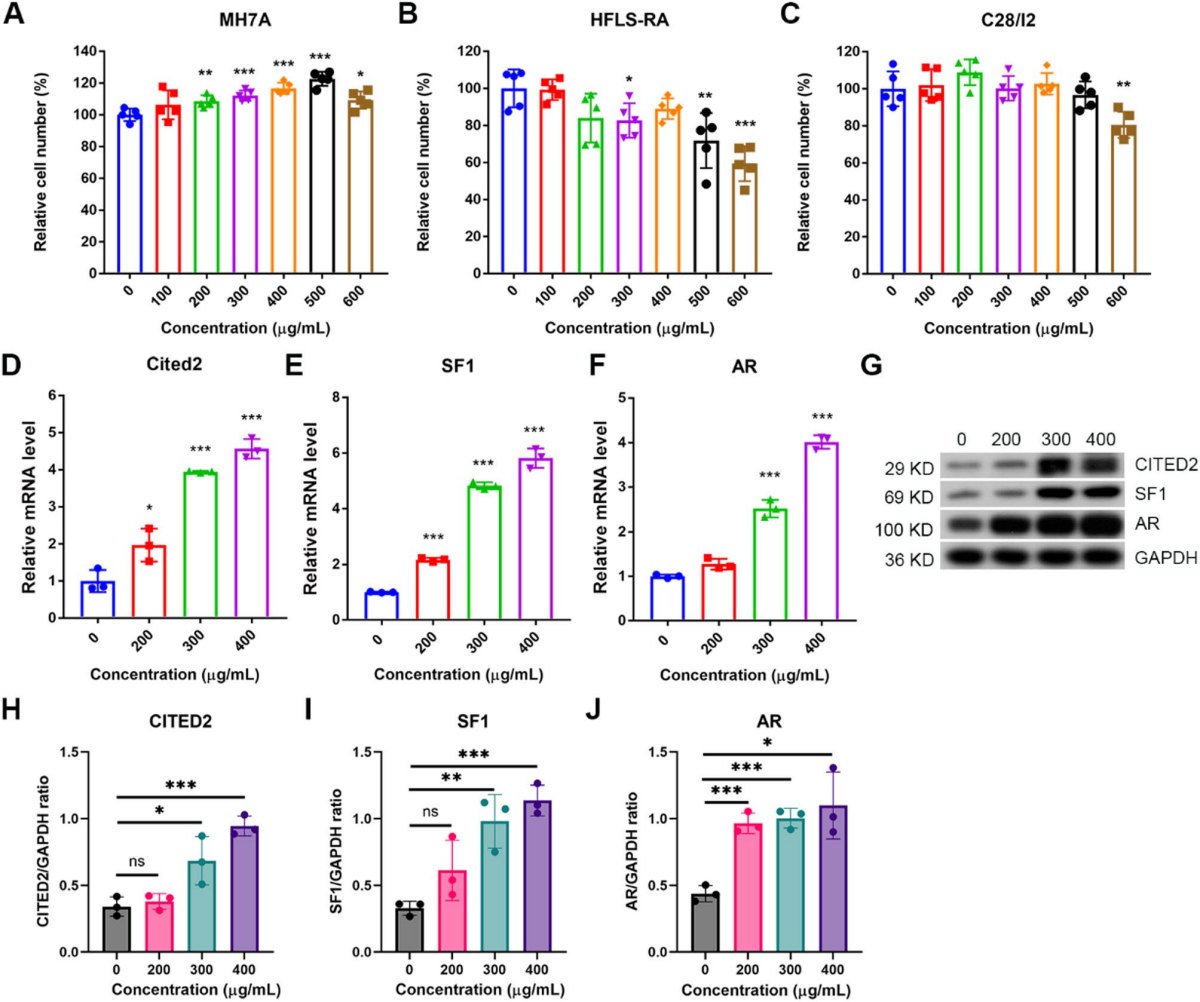
*A. mongholicus* and *S. divaricate* extract altered synoviocytes growth and up-regulated hub transcription factors expression. **A**, *A. mongholicus* and *S. divaricate* extract stimulated MH7A synoviocytes growth in a concentration-dependent manner. **B** and **C**, *A. mongholicus* and *S. divaricate* extract did not enhance synoviocytes HFLS-RA cells and C28/I2 chondrocytic cells growth. **D-F**, *A. mongholicus* and *S. divaricate* extract increased hub transcription factors CITED2, SF1, and AR expression on mRNA level in MH7A synoviocytes. **G**, *A. mongholicus* and *S. divaricate* extract increased Cited2, SF1, and AR expression on protein level in MH7A synoviocytes (Representative picture of 3 independent Western blots). **H**, Quantitation of the ratio of CITED2/GAPDH from 3 independent Western blots. **I**, Quantitation of the ratio of SF1/GAPDH from 3 independent Western blots. **J**, Quantitation of the ratio of AR/GAPDH from 3 independent Western blots. NS denotes not significant, * denotes *P* < 0.05, ** denotes *P* < 0.01 and *** denotes *P* < 0.001

We identified the three hub transcription factors and their target genes from all existing datasets. Whether *A. mongholicus* and *S. divaricata* have any effects on chondrocyte or synoviocyte gene expression has not been explored. We first investigated whether *A. mongholicus* and *S. divaricata* extracts have any effects on chondrocyte or synoviocyte growth. The HPLC-DAD analysis revealed that the *A. mongholicus* and *S. divaricata* extraction methods provided consistent active compounds and relatively equal amounts of the major compounds. Supplemental Fig. 3 shows that the major peaks of the HPLC-DAD chromatograms were almost the same. Interestingly, *A. mongholicus* and *S. divaricata* extracts stimulated the growth of MH7A synoviocytes at concentrations ranging from 300 µg/mL to 600 µg/mL (Fig. 5A), but did not promote the growth of HFLS-RA synoviocytes or C28/I2 chondrocytes (Fig. 5B-C). Cytotoxicity was observed at high concentrations (600 µg/mL) in both HFLS-RA synoviocytes and C28/I2 chondrocytes (Fig. 5B, C). We further examined whether *A. mongholicus* and *S. divaricata* extracts had any effects on the expression of the three hub transcription factors in MH7A synoviocytes. Based on the data showed in Fig. 5A-C, we used 200–400 µg/mL *A. mongholicus* and *S. divaricata* extracts for this set of experiments to prove this concept because no obvious cytotoxicity was observed in either MH7A synoviocytes or C28/I2 chondrocytes at this concentration. Interestingly, *A. mongholicus* and *S. divaricata* extracts increased the expression of all three hub transcription factor genes in MH7A synoviocytes (Fig. 5D-F) at the mRNA level. The upregulation of CITED2 in chondrocytes has been suggested to be beneficial in OA [49, 50]. We further validated the protein expression levels of the three hub transcription factors. Western blot analysis revealed that CITED2, SF1, and AR protein expression were indeed increased by *A. mongholicus* and *S. divaricata* extracts in MH7A synoviocytes (Fig. 5G and H).

### A. mongholicus and S. divaricata extracts reduced MMP2 and MMP9 expression in synoviocytes

**Fig. 6:**
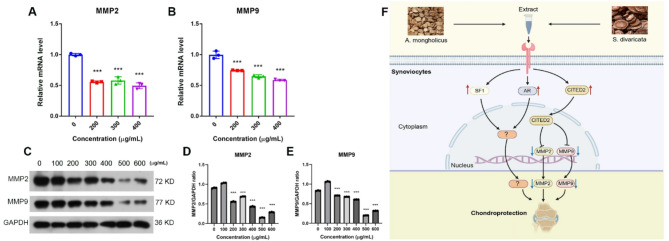
**A**. *mongholicus* and *S. divaricate* extract decreased MMP2 and MMP9 expression in human synoviocytes. A, *A. mongholicus* and *S. divaricate* extract decreased MMP2 expression on mRNA level in synoviocytes. **B**, *A. mongholicus* and *S. divaricate* extract decreased MMP9 expression on mRNA level in synoviocytes. **C**, *A. mongholicus* and *S. divaricate* extract down-regulated MMP2 and MMP9 expression on protein level in synoviocytes (Representative figure of 3 independent Western blots). **D**, Quantification of average MMP2/GAPDH ratio from 3 Western blots. **E**, Quantification of average MMP9/GAPDH from 3 Western blots. **F**, Schematic diagram of proposed mechanisms. *** denotes *P* < 0.001

Higher expression levels of MMP2 and MMP9 have been observed in human OA tissues [51]. Inhibiting MMP2 and MMP9 mitigates OA progression [52, 53]. Our bioinformatics analysis revealed that MMP2 and MMP9 are two of the hub genes and target genes of the hub transcription (co-)factors. We thus hypothesized that *A. mongholicus* and *S. divaricata* extracts decrease the expression of the cell matrix-degrading enzymes MMP2 and MMP9 in synoviocytes. We tested this hypothesis by treating MH7A and HFLS-RA synoviocytes with *A. mongholicus* and *S. divaricata* extracts. Interestingly, MMP2 and MMP9 mRNA expression in MH7A synoviocytes did not respond to *A. mongholicus* or *S. divaricata* extract treatment (data not shown). However, the mRNA expression levels of MMP2 and MMP9 in HFLS-RA synoviocytes were significantly reduced by *A. mongholicus* and *S. divaricata* extract treatment (Fig. 6A, B). The downregulation of both the MMP2 and MMP9 proteins was observed in HFLS-RA synoviocytes (Fig. 6C, D, E).

Based on the experimental data, we hypothesize that *Astragalus mongholicus* and *Saposhnikovia divaricata* extracts confer chondroprotective effects via modulation of key transcriptional regulators (Fig. 6F). Treatment with these botanical extracts upregulated the expression of CITED2, SF1, and AR in synoviocytes. CITED2 is known to suppress the transcription and secretion of matrix metalloproteinases MMP2 and MMP9, thereby potentially attenuating cartilage matrix degradation. While the roles of SF1 and AR in chondroprotection are increasingly recognized, the precise molecular pathways through which they mediate these effects remain to be elucidated and merit further investigation.

## Discussion

Osteoarthritis (OA) is a disease of the whole joint that involves bone, cartilage, ligaments, the fat pad, and the synovium. No cure is available for OA thus far. The identification of therapeutic targets and the underlying mechanisms are essential for finding a cure or providing relief of OA symptoms. The synovium plays a critical role in early OA pathogenesis and disease progression. Identifying therapeutic targets in the synovium or synoviocytes may provide insights into OA symptom relief, prevention, or even a cure. Traditional Chinese medicine has the potential to reduce inflammation and relieve OA symptoms by downregulating the levels of the proinflammatory cytokines IL-1β, IL-6, IL-17, and TNF-α, as well as MMPs (such as MMP-2 and MMP-9), in animal models [54–56]. Consistent with previous reports, we observed that *A. mongholicus* and *S. divaricata* extracts downregulated proinflammatory cytokine and MMP expression in human synoviocytes. *A. mongholicus* and *S. divaricata* have been reported to relieve OA symptoms; however, the underlying mechanisms are not known. Because of the extreme complexity of the active compounds of *A. mongholicus* and *S. divaricata*, a manual search for compounds and their potential target genes is nearly impossible. We utilized an in silico database search method, which allowed us to obtain the potential targets of the drug pair. The advantages of gene screening methods include high-throughput screening, relatively efficient screening, and the identification of genes with therapeutic potential. We identified hub genes and hub transcription factors via this method. In this study, we identified three hub transcription factors (CITED2, AR, and SF1) whose target gene expression was altered by *A. mongholicus* and *S. divaricata* in the synovium. CITED2 inhibits the expression of matrix metallopeptidases (such as MMP1 and MMP13) in chondrocytes [49, 50]. CITED2 can also act as a regulator of C-C motif chemokine ligand (CCL) expression in cancer cells [57]. Therefore, the active compounds from *A. mongholicus* and *S. divaricata* may increase CITED2 expression, subsequently suppress C-C motif chemokine ligand expression and reduce proinflammatory cytokine and MMP expression, which exerts chondroprotective effects, as observed. However, we are planning further investigations to determine whether this hypothesis is correct, and we will report the results elsewhere.

The androgen receptor functions as a steroid hormone-activated transcription factor. The AR gene has been reported to be associated with the risk of OA [58]. Interestingly, in other organs or tissues, activation of the androgen receptor pathway exerts many anti-inflammatory and protective effects via a variety of pathways and mechanisms [59]. We observed that *A. mongholicus* and *S. divaricata* extracts could increase AR expression in synoviocytes, which could lead to anti-inflammatory and protective effects on joints. However, whether AR pathways play any anti-inflammatory role in synoviocytes or chondrocytes and thus play any role in OA and the underlying mechanisms are not known. Further investigations are needed to elucidate the roles of AR pathways in OA and the underlying mechanisms involved.

Differential growth response of cell lines was observed in this study, namely only MH7A synoviocytes growth was enhanced. The possible reasons could be the intrinsic cellular differences among three cell types, as well as the variations in their signaling pathways, receptor or transporter expression, and metabolic profiles. For example, MH7A cells are synoviocytes, whereas C28/I2 are chondrocytes, they may have distinct growth requirements and responses to herbal compounds.

This study has several limitations. First, this study utilized available public databases, and these databases have limitations. One of the advantages of the gene screening method is that it is a high-throughput technology and allows us to identify potential therapeutic targets and signaling pathways more efficiently and accurately. On the other hand, the outcome of the search relies on databases, which could be incomplete or inaccurate. Future studies should incorporate curated, updated datasets, and where feasible, integrate multi-omics approaches (e.g., transcriptomics, proteomics, metabolomics) to validate and refine bioinformatic predictions. Second, this study focused on the identification of hub genes and hub transcription factors in human synoviocytes. To strengthen the biological relevance of these findings, expanded validation using primary human synoviocytes and chondrocytes, as well as in vivo models (such as mouse or rat OA model), is warranted. Such approaches would provide more comprehensive insights into the chondroprotective effects of *Astragalus mongholicus* and *Saposhnikovia divaricata* extracts. Third, putative active compounds were obtained from the TCMID and TCMSP databases. The oral bioavailability and drug-likeness values are based on computational predictions and may not always accurately reflect the actual biological activity or therapeutic potential of a compound in vivo. The OB/DL criteria provided by the TCMSP could be subjective and variable or even controversial [60, 61]. This limitation highlights one of the reasons we validated the biological response of synoviocytes to the two herb extracts.

Future research should include experimental validation of compound bioactivity, pharmacokinetic profiling, and structure-activity relationship (SAR) analyses. Moreover, a systematic and transparent compound selection strategy, possibly incorporating machine learning or network pharmacology, could improve the accuracy and reproducibility of candidate identification.

## Conclusion

Taken together, the results of this study revealed hub genes and hub transcription factors that *A. mongholicus* and *S. divaricata* can alter in synoviocytes. The hub genes could serve as OA biomarkers, and the hub transcription factors (co-factors) could serve as targets for OA symptom relief or therapeutic interventions.

## Supplementary Information

Below is the link to the electronic supplementary material.

Supplementary Material 1

Supplementary Material 2

Supplementary Material 3

Supplementary Material 4

Supplementary Material 5

Supplementary Material 6

Supplementary Material 7

Supplementary Material 8

## Data Availability

The data generated and/or analyzed during the current study are available from the corresponding author on reasonable request.

## Abbreviations

Abbreviation: Full Term
OA: Osteoarthritis
TCM: Traditional Chinese Medicine
DEGs: Differentially Expressed Genes
GO: Gene Ontology
KEGG: Kyoto Encyclopedia of Genes and Genomes
PPI: Protein–Protein Interaction
TF: Transcription Factor
AR: Androgen Receptor
CITED2: CBP/p300-interacting transactivator with Glu/Asp-rich carboxy-terminal domain 2
SF1: Splicing Factor 1
MMP2: Matrix Metallopeptidase 2
MMP9: Matrix Metallopeptidase 9
PCR: Polymerase Chain Reaction
qPCR: Quantitative PCR
GAPDH: Glyceraldehyde-3-Phosphate Dehydrogenase
NC: Nitrocellulose (membrane)
DAD: Diode Array Detector
HPLC: High-Performance Liquid Chromatography
ROC: Receiver Operating Characteristic
AUC: Area Under the Curve
TLR: Toll-Like Receptor
IL: Interleukin
TNF-α: Tumor Necrosis Factor-alpha
TGF-β: Transforming Growth Factor-beta
NF-κB: Nuclear Factor kappa-light-chain-enhancer of activated B cells
OB: Oral Bioavailability
DL: Drug-Likeness
TCMSP: Traditional Chinese Medicine Systems Pharmacology Database
TCMID: Traditional Chinese Medicines Integrated Database
OMIM: Online Mendelian Inheritance in Man
GEO: Gene Expression Omnibus
FDR: False Discovery Rate
