# Supplementary material for: Identification of target genes of Astragalus mongholicus and Saposhnikovia divaricata extracts in human synoviocytes for potential osteoarthritis treatment
Source: Hereditas. 2025 Oct 8;162:203. doi: 10.1186/s41065-025-00581-7 (PMC12506284; doi:10.1186/s41065-025-00581-7)
Supplement: Supplementary file 1 — Supplementary Material 1 [file 41065_2025_581_MOESM1_ESM.docx]

**Supplemental Table 1. Primer sequences and PCR product sizes**

| **Gene name** | **Forward primer** | **Reverse primer** | **PCR product size** |
| --- | --- | --- | --- |
| GAPDH | ACAACTTTGGTATCGTGGAAGG | GCCATCACGCCACAGTTTC | 101 |
| CITED2 | AACCAGCACTTCCGAGATTGC | AATCAGTGTCTATGACATTGGGC | 217 |
| AR | GACGACCAGATGGCTGTCATT | GGGCGAAGTAGAGCATCCT | 106 |
| SF1 | AAGGTTGGGCGCAAAGATG | ACGTTCTCCATTGTATTGGCAG | 86 |
| MMP9 | GGGACGCAGACATCGTCATC | TCGTCATCGTCGAAATGGGC | 139 |
| MMP2 | CCCACTGCGGTTTTCTCGAAT | CAAAGGGGTATCCATCGCCAT | 89 |
