# Supplementary material for: Identification of target genes of Astragalus mongholicus and Saposhnikovia divaricata extracts in human synoviocytes for potential osteoarthritis treatment
Source: Hereditas. 2025 Oct 8;162:203. doi: 10.1186/s41065-025-00581-7 (PMC12506284; doi:10.1186/s41065-025-00581-7)
Supplement: Supplementary file 2 — Supplementary Material 2 [file 41065_2025_581_MOESM2_ESM.doc]

| **Supplemental Table 2: The characteristics of potential active ingredients** | | | | | | |  |  |
| --- | --- | --- | --- | --- | --- | --- | --- | --- |
| Compounds | Molecular formula | Structure | Molecular weight | OB(%) | DL | HL | From | Classification |
| Jaranol | 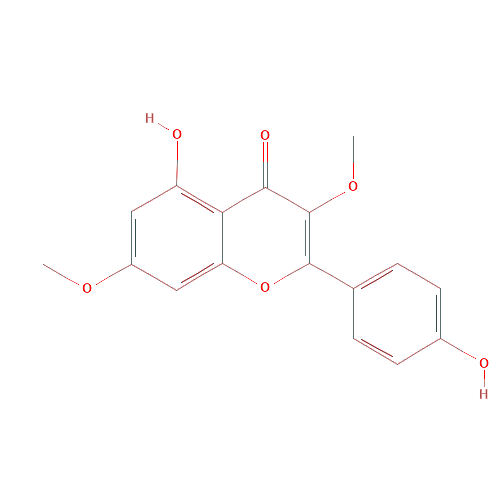C17H14O6 |  | 314.29 | 50.83 | 0.29 | 15.50 | Huangqi | Flavone |
| isorhamnetin | 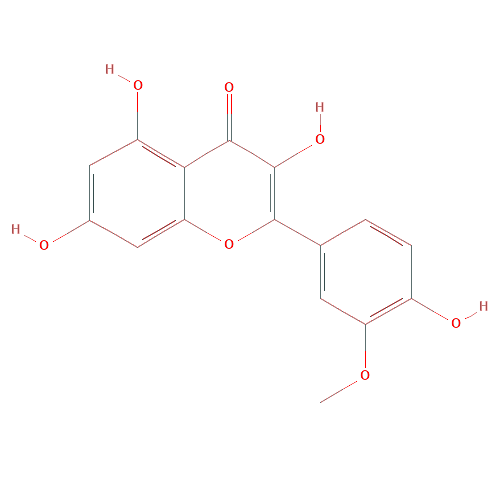C16H12O7 | |  | | --- | | 316.26 | 49.6 | 0.31 | 14.34 | Huangqi | Flavone |
| Calycosin | C16H12O5 | 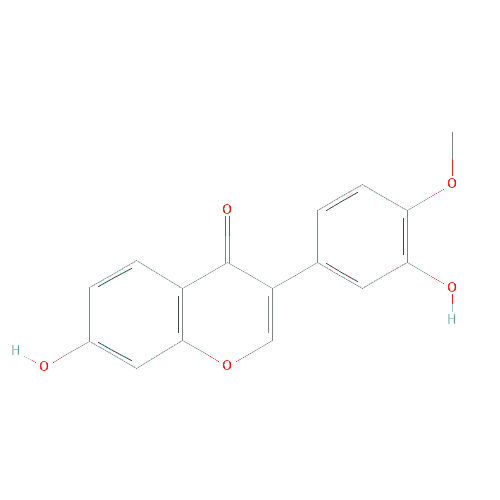 | 284.26 | 47.75 | 0.24 | 17.1 | Huangqi | Flavone |
| kaempferol | C15H10O6 | 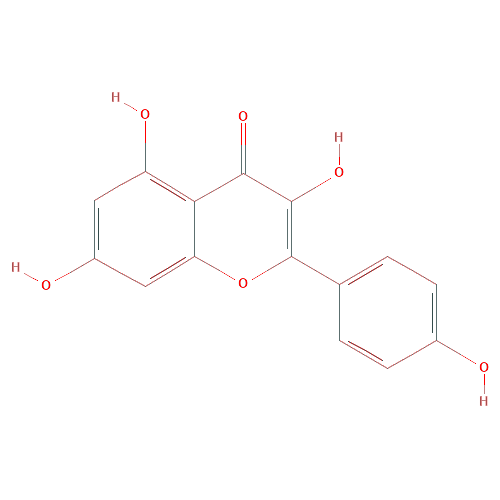 | 286.24 | 41.88 | 0.24 | 14.74 | Huangqi | Flavone |
| Bifendate | C20H18O10 | | 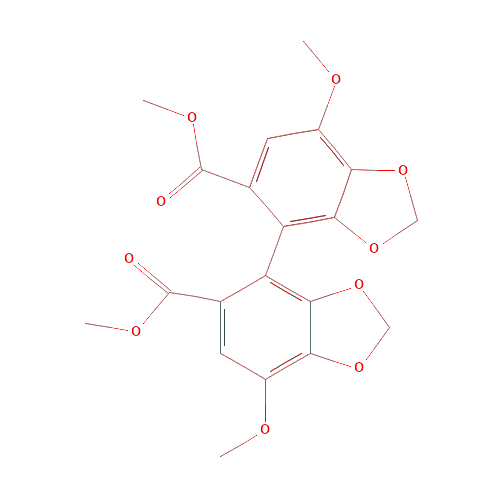 | | --- | | 418.3 | 31.1 | 0.67 | 17.96 | Huangqi | Lactone |
| 3,9-di-O-methylnissolin | C18H18O5 | 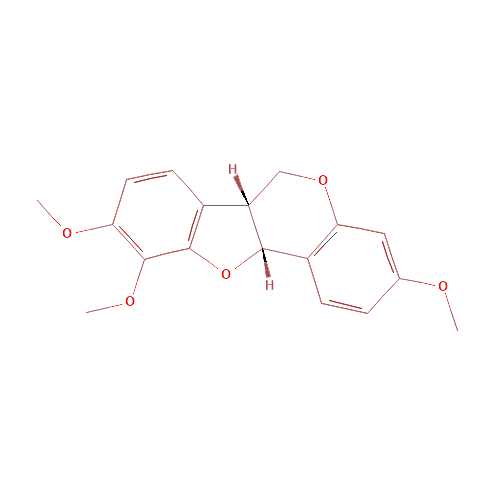 | 314.3 | 53.74 | 0.48 | 9 | Huangqi | Ethers |
| 7-O-methylisomucronulatol | C18H20O5 | 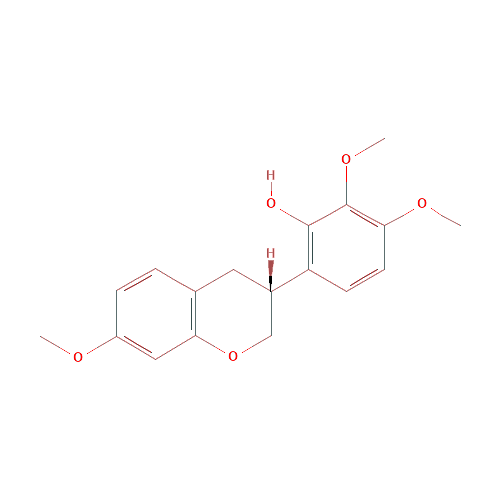 | 316.3 | 74.69 | 0.3 | 2.98 | Huangqi | Ethers |
| (3R)-3-(2-hydroxy-3,4-dimethoxyphenyl)chroman-7-ol | | C17H18O5 | | --- | | 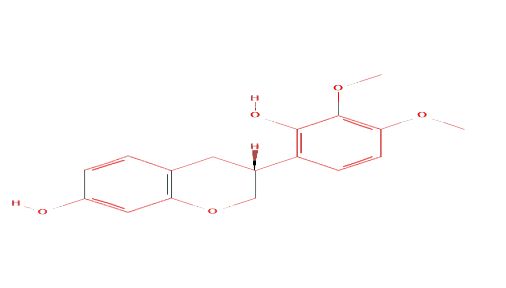 | 302.32 | 67.67 | 0.26 | 2.9 | Huangqi | Ethers |
| 1,7-Dihydroxy-3,9-dimethoxy pterocarpene | C17H14O6 | 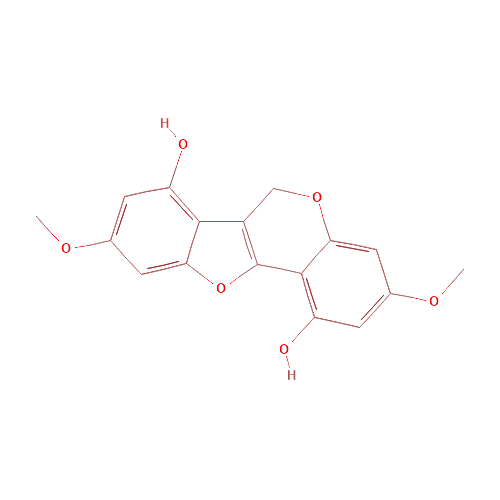 | 314.29 | 39.05 | 0.48 | 7.95 | Huangqi | Ethers |
| Mairin | C30H48O3 | 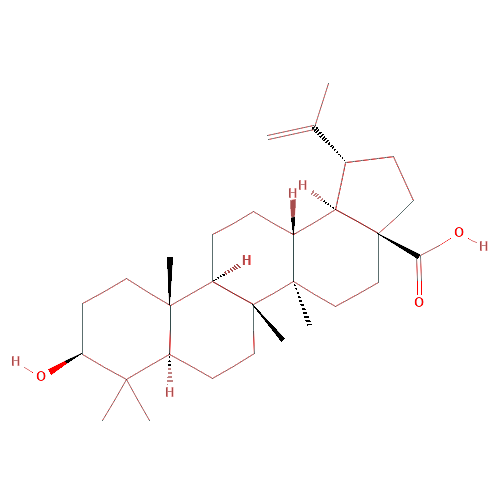 | 456.7 | 55.38 | 0.78 | 8.87 | Huangqi | Pentacyclic triterpenes |
| hederagenin | C30H48O4 | 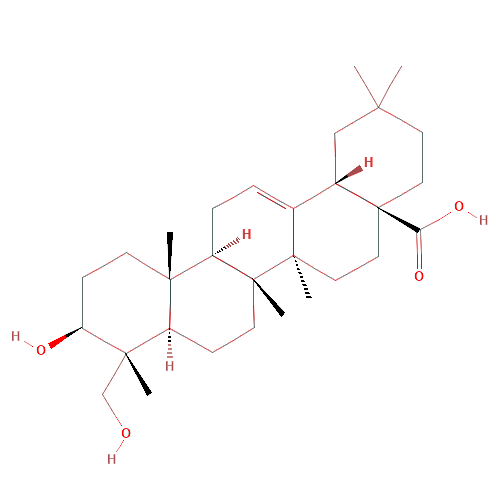 | 472.7 | 36.91 | 0.75 | 5.35 | Huangqi | Pentacyclic triterpenes |
| quercetin | C15H10O7 | 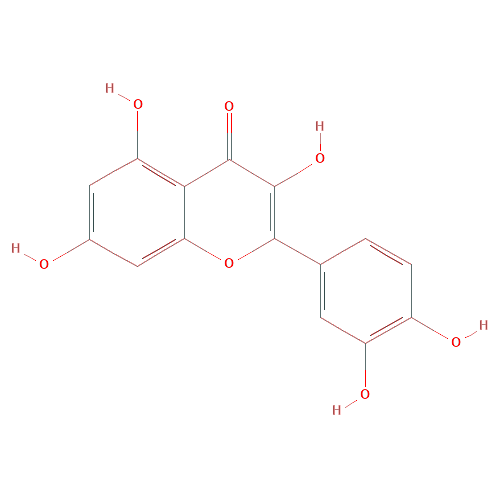 | 302.23 | 46.43 | 0.28 | 14.4 | Huangqi | Flavone |
| formononetin | C16H12O4 | 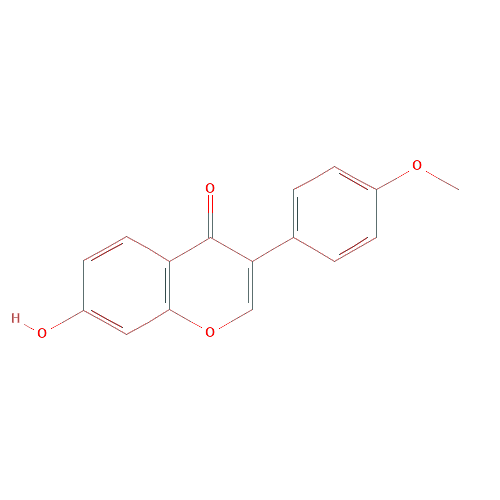 | 268.26 | 69.67 | 0.21 | 17.04 | Huangqi | Flavone |
| isomucronulatol-7,2'-di-O-glucosiole | C29H38O15 | 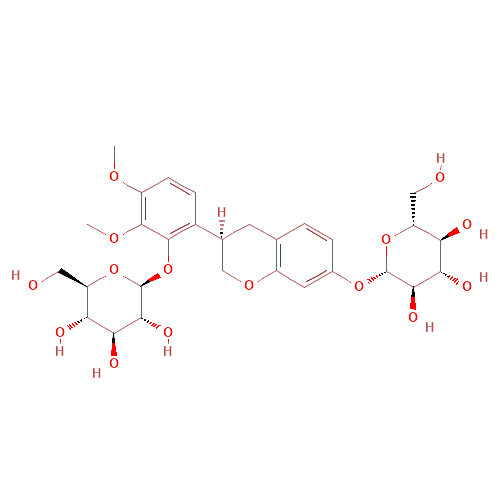 | 626.6 | 49.28 | 0.62 | 0.93 | Huangqi | Glycosides |
| FA | C19H19N7O6 | | 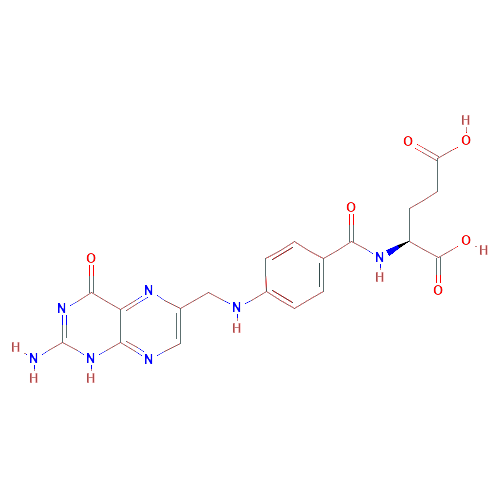 | | --- | | 441.4 | 68.96 | 0.71 | 24.81 | Huangqi | Alkaloids |
| isoflavanone | C15H12O2 | 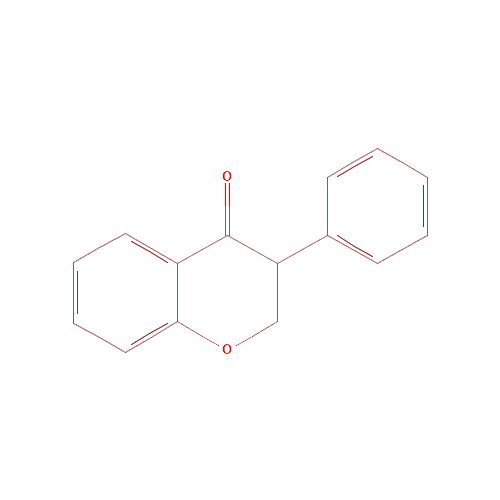 | 224.25 | 109.99 | 0.3 | 15.51 | Huangqi | Flavone |
| (3S,8S,9S,10R,13R,14S,17R)-10,13-dimethyl-17-[(2R,5S)-5-propan-2-yloctan-2-yl]-2,3,4,7,8,9,11,12,14,15,16,17-dodecahydro-1H-cyclopenta[a]phenanthren-3-ol | C30H52O | 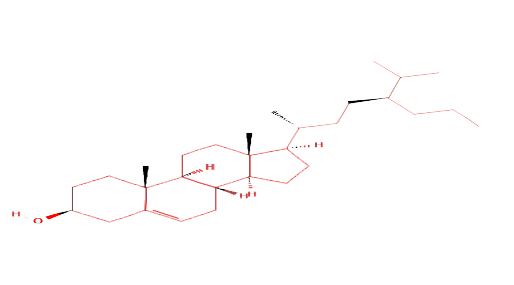 | 428.7 | 36.23 | 0.78 | 5.22 | Huangqi | Steroids |
| sitosterol | C29H50O | 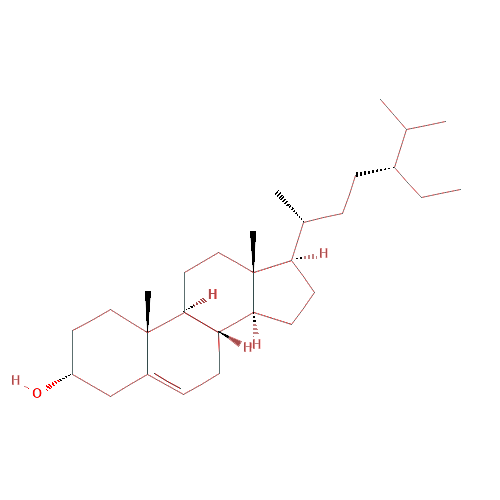 | 414.7 | 36.91 | 0.75 | 5.37 | Fangfeng | Steroids |
| Phellopterin | C17H16O5 | 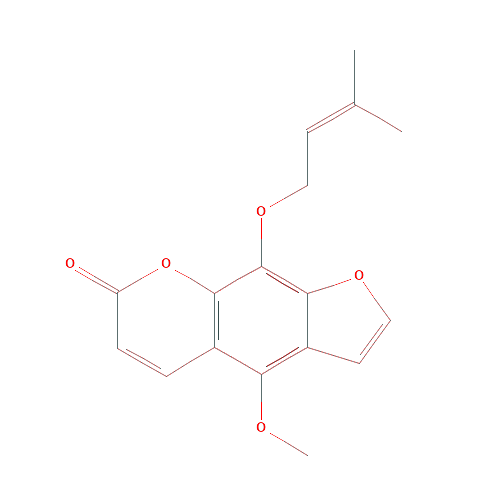 | 300.3 | 40.19 | 0.28 | -1.94 | Fangfeng | Lactone |
| Ammidin | C16H14O4 | 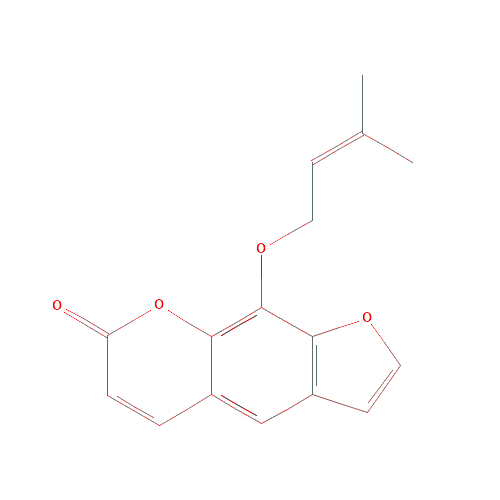 | 270.28 | 34.55 | 0.22 | -1.35 | Fangfeng | Lactone |
| anomalin | C24H26O7 | 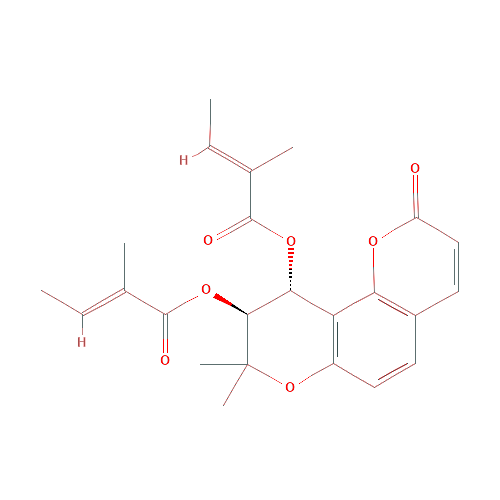 | 426.5 | 59.65 | 0.66 | 1.59 | Fangfeng | Lactone |
| 5-O-Methylvisamminol | C16H18O5 | 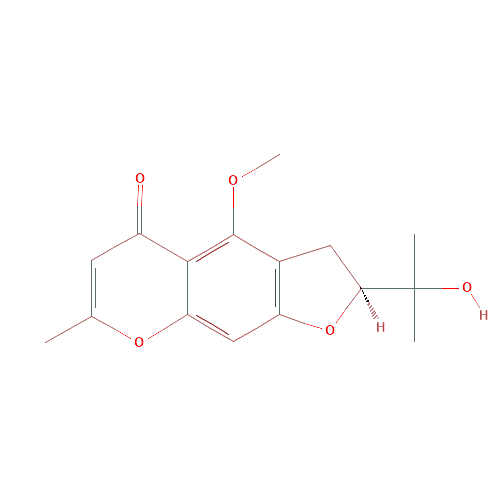 | 290.31 | 37.99 | 0.25 | 14.67 | Fangfeng | Ethers |
| wogonin | C16H12O5 | 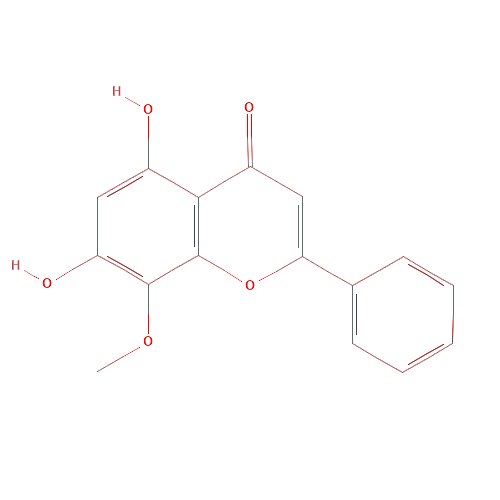 | 284.26 | 30.68 | 0.23 | 17.75 | Fangfeng | Flavone |
| (2R,3R)-3-(4-hydroxy-3-methoxy-phenyl)-5-methoxy-2-methylol-2,3-dihydropyrano[5,6-h][1,4]benzodioxin-9-one | C20H18O8 | 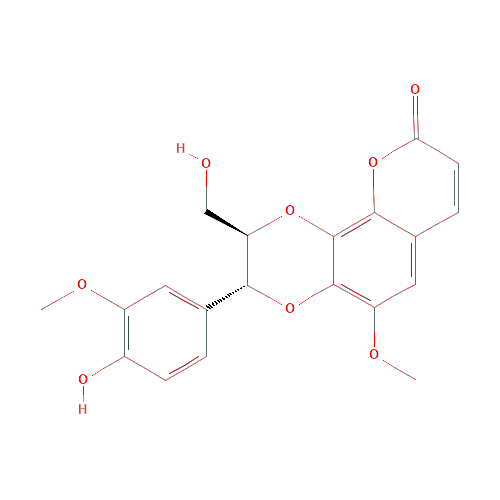 | 386.4 | 68.83 | 0.66 | 3.39 | Fangfeng | Lactone |
| Prangenidin | C16H14O4 | 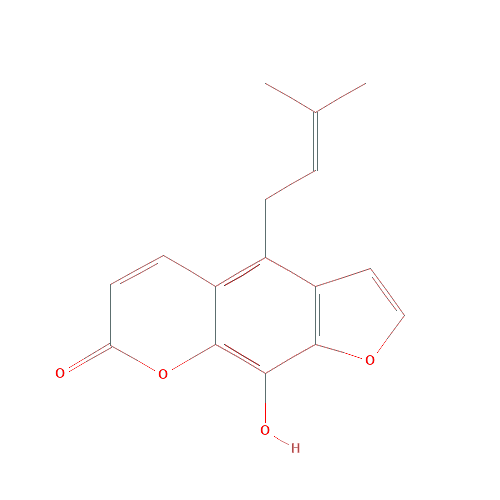 | 270.28 | 36.31 | 0.22 | -2.39 | Fangfeng | Lactone |
| beta-sitosterol | C29H50O | | 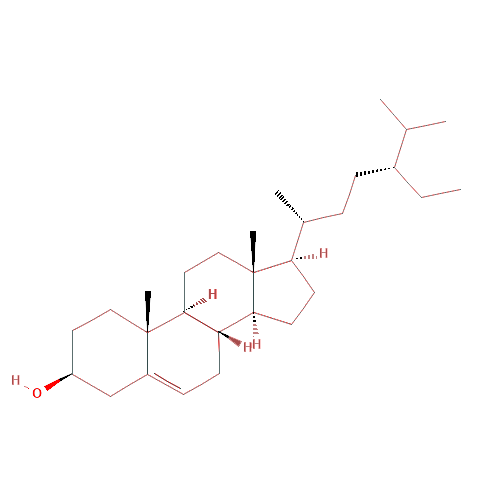 | | --- | | 414.7 | 36.91 | 0.75 | 5.36 | Fangfeng | Steroids |
| Decursin | C19H20O5 | 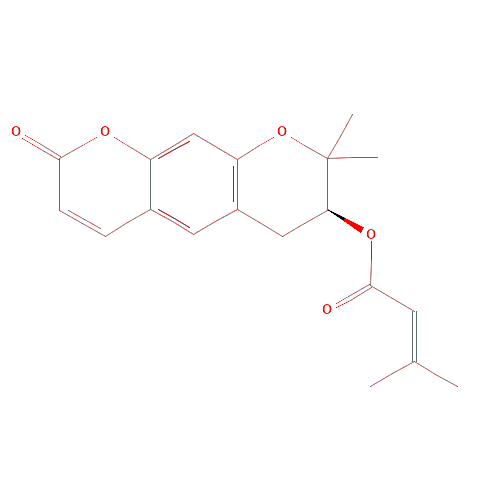 | 328.4 | 39.27 | 0.38 | -1.77 | Fangfeng | Lactone |
| isoimperatorin | C16H14O4 | | 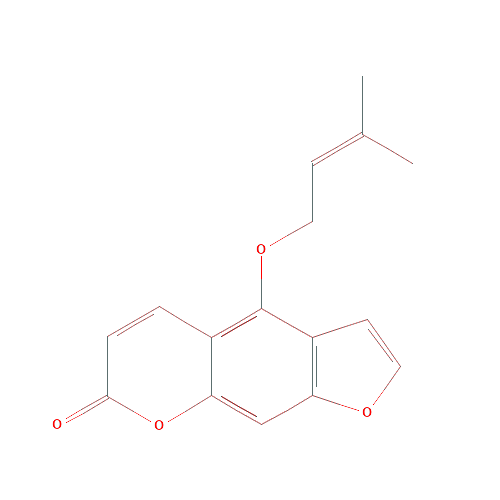 | | --- | | 270.28 | 45.46 | 0.23 | -1.44 | Fangfeng | Lactone |
