# Supplementary material for: Identification of target genes of Astragalus mongholicus and Saposhnikovia divaricata extracts in human synoviocytes for potential osteoarthritis treatment
Source: Hereditas. 2025 Oct 8;162:203. doi: 10.1186/s41065-025-00581-7 (PMC12506284; doi:10.1186/s41065-025-00581-7)
Supplement: Supplementary file 3 — Supplementary Material 3 [file 41065_2025_581_MOESM3_ESM.docx]

| **Supplemental Table 3: The characteristics of 58 targets.** | | | | |
| --- | --- | --- | --- | --- |
| **Target** | **Name** | **Degree** | **Betweenness centrality** | **Closeness centrality** |
| ALB | Serum albumin | 48 | 0.09510307 | 0.86363636 |
| CCND1 | G1/S-specific cyclin-D1 | 40 | 0.04475443 | 0.77027027 |
| HSP90AA1 | Heat shock protein HSP 90-alpha | 40 | 0.05227192 | 0.77027027 |
| CTNNB1 | Catenin beta-1 | 39 | 0.03227109 | 0.76 |
| ERBB2 | Receptor tyrosine-protein kinase erbB-2 | 38 | 0.03686468 | 0.74025974 |
| AR | Androgen receptor | 38 | 0.04626097 | 0.75 |
| MMP9 | Matrix metalloproteinase-9 | 38 | 0.0311475 | 0.74025974 |
| MMP2 | 72 kDa type IV collagenase | 36 | 0.02634192 | 0.72151899 |
| APP | Amyloid beta A4 protein | 36 | 0.02719111 | 0.72151899 |
| SOD2 | Superoxide dismutase [Mn], mitochondrial | 33 | 0.02827068 | 0.69512195 |
| MDM2 | E3 ubiquitin-protein ligase Mdm2 | 32 | 0.01843393 | 0.69512195 |
| MAPK9 | Mitogen-activated protein kinase 9 | 31 | 0.02849075 | 0.68674699 |
| F2 | Prothrombin | 31 | 0.01936997 | 0.68674699 |
| ITPKC | Inositol-trisphosphate 3-kinase C | 31 | 0.0138204 | 0.68674699 |
| B2M | Beta-2-microglobulin | 30 | 0.01518046 | 0.67058824 |
| ALK | ALK tyrosine kinase receptor | 26 | 0.0403158 | 0.64044944 |
| S100A8 | Protein S100-A8 | 26 | 0.01443222 | 0.63333333 |
| NCAM1 | Neural cell adhesion molecule 1 | 24 | 0.00525006 | 0.62637363 |
| TGM2 | Protein-glutamine gamma-glutamyltransferase 2 | 23 | 0.00460886 | 0.61290323 |
| MMP14 | Matrix metalloproteinase-14 | 22 | 0.00501725 | 0.60638298 |
| MAP2K2 | Dual specificity mitogen-activated protein kinase kinase | 22 | 0.01445495 | 0.61290323 |
| GZMB | Granzyme B | 22 | 0.00454124 | 0.60638298 |
| HDAC6 | Histone deacetylase 6 | 21 | 0.00504323 | 0.61290323 |
| RAC1 | Ras-related C3 botulinum toxin substrate 1 | 21 | 0.01305142 | 0.60638298 |
| ADAM17 | ADAM 17 | 20 | 0.00132395 | 0.59375 |
| PCSK9 | Proprotein convertase subtilisin/kexin type 9 | 20 | 0.00663897 | 0.6 |
| DDB1 | DNA damage-binding protein 1 | 19 | 0.03149226 | 0.59375 |
| HFE | Hereditary hemochromatosis protein | 18 | 0.00411404 | 0.58163265 |
| E2F1 | Transcription factor E2F1 | 18 | 0.00295358 | 0.59375 |
| PCNA | Proliferating cell nuclear antigen | 17 | 0.00807728 | 0.57575758 |
| PDPK1 | 3-phosphoinositide-dependent protein kinase 1 | 16 | 0.00078866 | 0.57575758 |
| IL6ST | Interleukin-6 receptor subunit beta | 16 | 0.00170033 | 0.55882353 |
| DDX58 | Probable ATP-dependent RNA helicase DDX58 | 15 | 0.0026268 | 0.57 |
| MAP3K3 | Mitogen-activated protein kinase kinase kinase 3 | 15 | 0.00475072 | 0.57575758 |
| DSP | Desmoplakin | 15 | 0.00375029 | 0.56435644 |
| INHBA | Inhibin beta A chain | 15 | 0.00364433 | 0.56435644 |
| ANXA3 | Annexin A3 | 14 | 0.00207064 | 0.55339806 |
| IFNGR1 | Interferon-gamma receptor alpha chain | 14 | 0.00165631 | 0.54807692 |
| F13A1 | Coagulation factor XIII A chain | 13 | 0.00150586 | 0.55339806 |
| LTF | Lactotransferrin | 13 | 0.00142026 | 0.54285714 |
| ASS1 | Argininosuccinate synthase | 13 | 0.00541601 | 0.55339806 |
| EIF2AK2 | Interferon-induced, double-stranded RNA-activated protein kinase | 12 | 0.00192313 | 0.55339806 |
| RND1 | Rho-related GTP-binding protein Rho6 | 11 | 0.00036449 | 0.54285714 |
| CHAT | Choline O-acetyltransferase | 11 | 0.00280483 | 0.54285714 |
| RARB | Retinoic acid receptor beta | 11 | 0.00066369 | 0.53773585 |
| GC | Vitamin D-binding protein | 10 | 0.000949 | 0.52777778 |
| SULT2A1 | Bile salt sulfotransferase | 9 | 0.00066813 | 0.52777778 |
| NR1I3 | Nuclear receptor subfamily 1 group I member 3 | 8 | 0.00048967 | 0.52293578 |
| GPD1L | Glycerol-3-phosphate dehydrogenase 1-like protein | 8 | 0.00238584 | 0.50442478 |
| USP14 | Ubiquitin carboxyl-terminal hydrolase 14 | 7 | 0.00007953 | 0.51351351 |
| FAP | Seprase | 7 | 0.00015959 | 0.50442478 |
| PRPS1 | Ribose-phosphate pyrophosphokinase 1 | 7 | 0.00377336 | 0.51351351 |
| CITED2 | Cbp/p300-interacting transactivator 2 | 7 | 0.00004177 | 0.51351351 |
| AMD1 | S-adenosylmethionine decarboxylase proenzyme | 6 | 0.00758761 | 0.51818182 |
| SF1 | Splicing factor 1 | 5 | 0.00506926 | 0.50892857 |
| VPS4B | Vacuolar protein sorting-associated protein 4B | 4 | 0.00048783 | 0.4453125 |
| PUF60 | Poly(U)-binding-splicing factor PUF60 | 3 | 0.00046992 | 0.39041096 |
| CLIC2 | Chloride intracellular channel protein 2 | 1 | 0 | 0.39310345 |
