# Supplementary figures and images for: Identification of target genes of Astragalus mongholicus and Saposhnikovia divaricata extracts in human synoviocytes for potential osteoarthritis treatment
Source: Hereditas. 2025 Oct 8;162:203. doi: 10.1186/s41065-025-00581-7 (PMC12506284; doi:10.1186/s41065-025-00581-7)

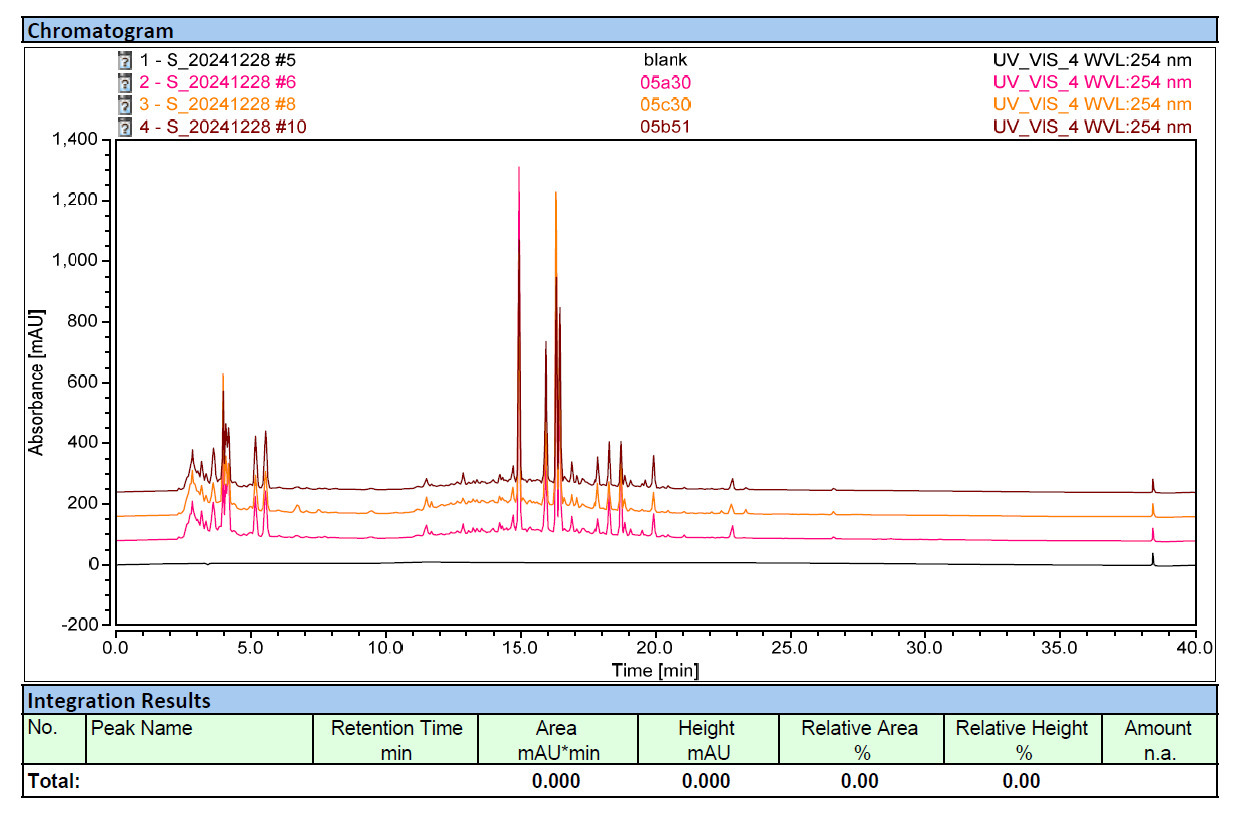

Supplement: Supplementary file 5 — Supplementary Material 5 [file 41065_2025_581_MOESM5_ESM.tif]

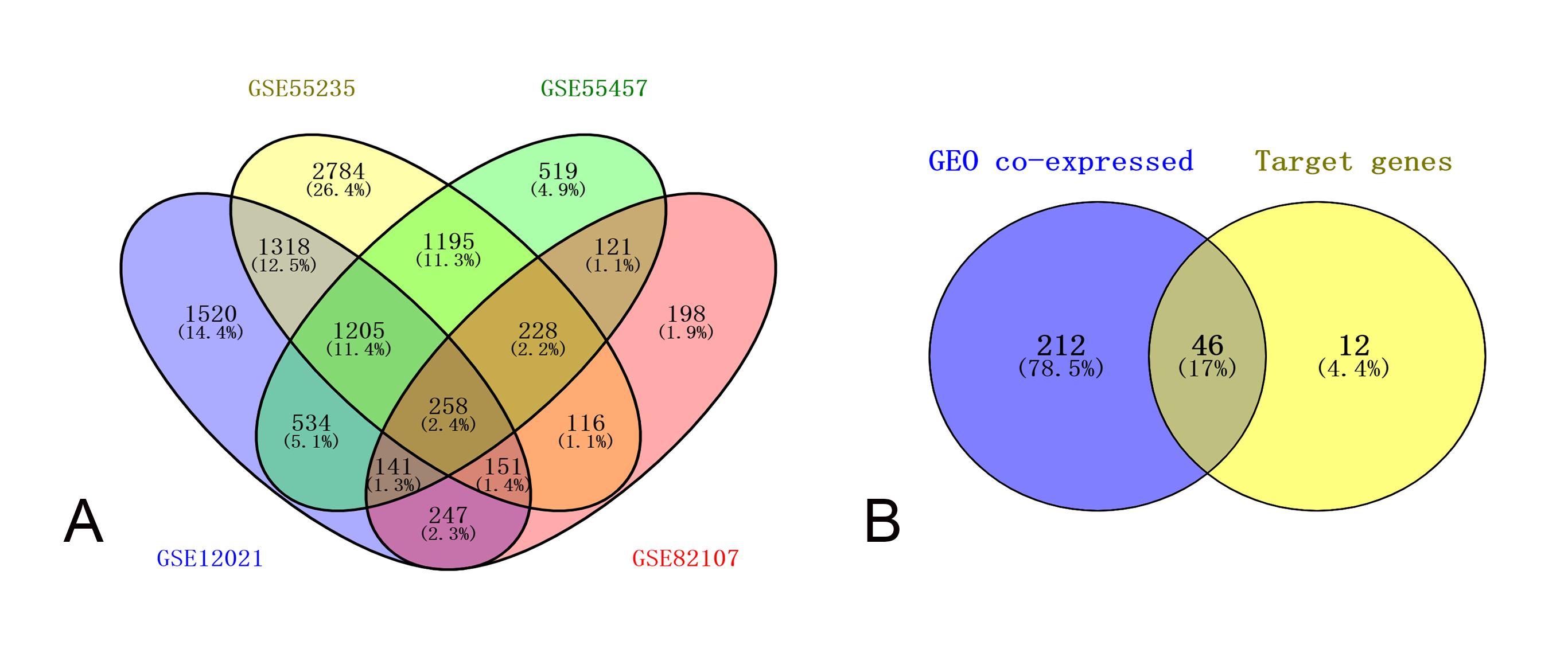

Supplement: Supplementary file 6 — Supplementary Material 6 [file 41065_2025_581_MOESM6_ESM.tif]

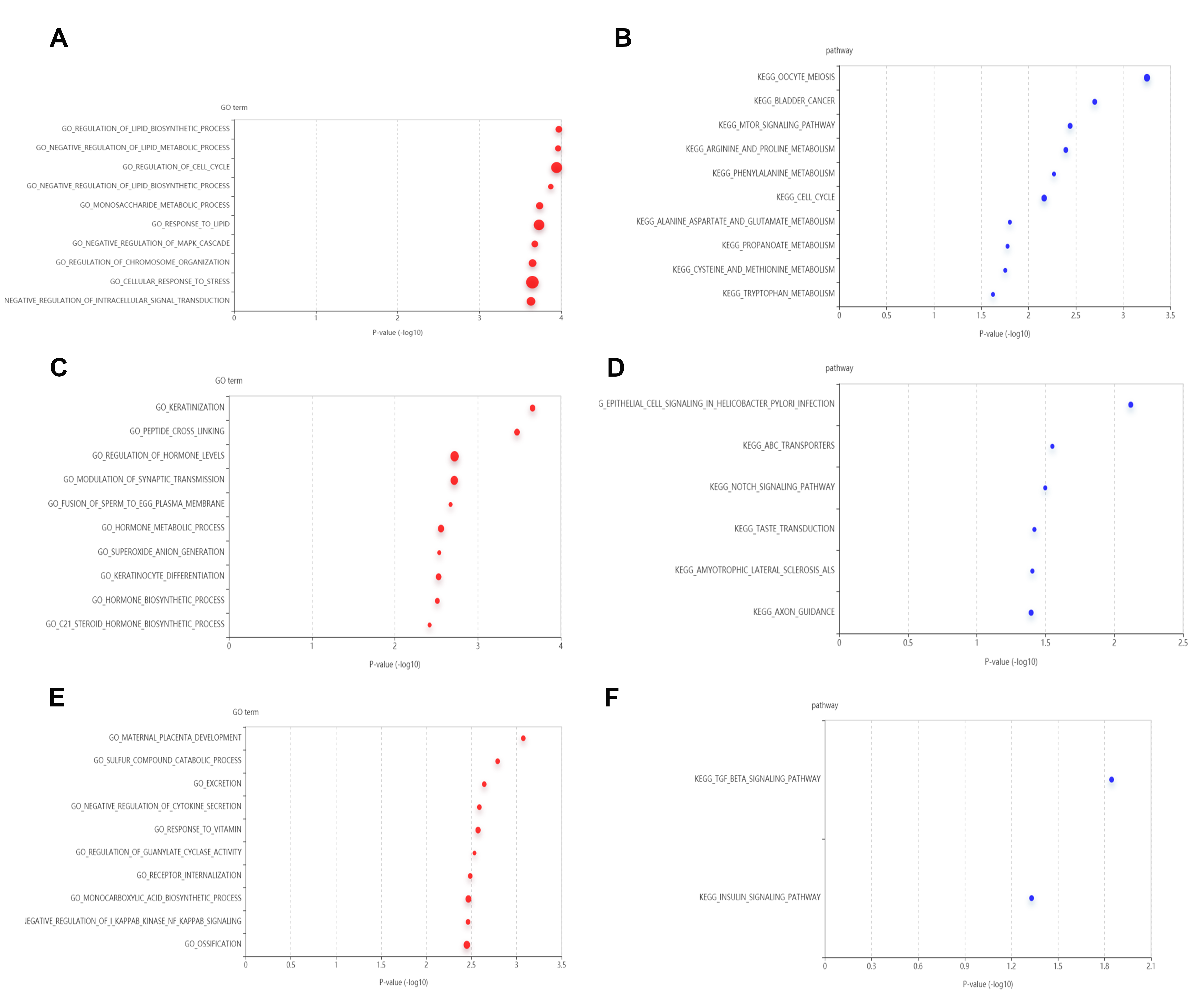

Supplement: Supplementary file 7 — Supplementary Material 7 [file 41065_2025_581_MOESM7_ESM.tif]
