# Supplementary material for: Identification of target genes of Astragalus mongholicus and Saposhnikovia divaricata extracts in human synoviocytes for potential osteoarthritis treatment
Source: Hereditas. 2025 Oct 8;162:203. doi: 10.1186/s41065-025-00581-7 (PMC12506284; doi:10.1186/s41065-025-00581-7)

Raw Western blots

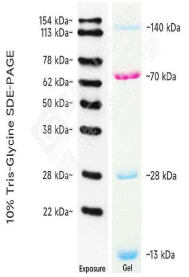

MMP2-L.E

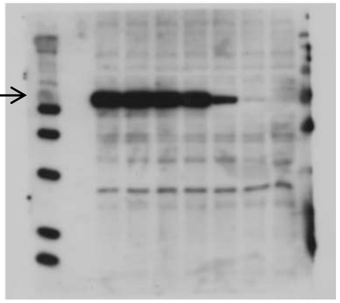

MMP2-S.E

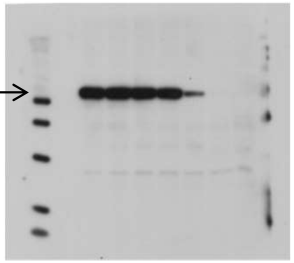

GAPDH-L.E

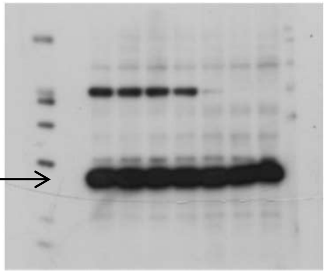

GAPDH -S.E

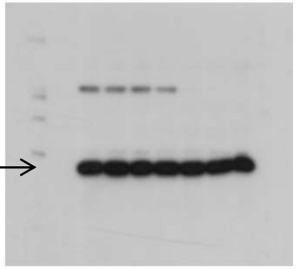

Raw Western blots

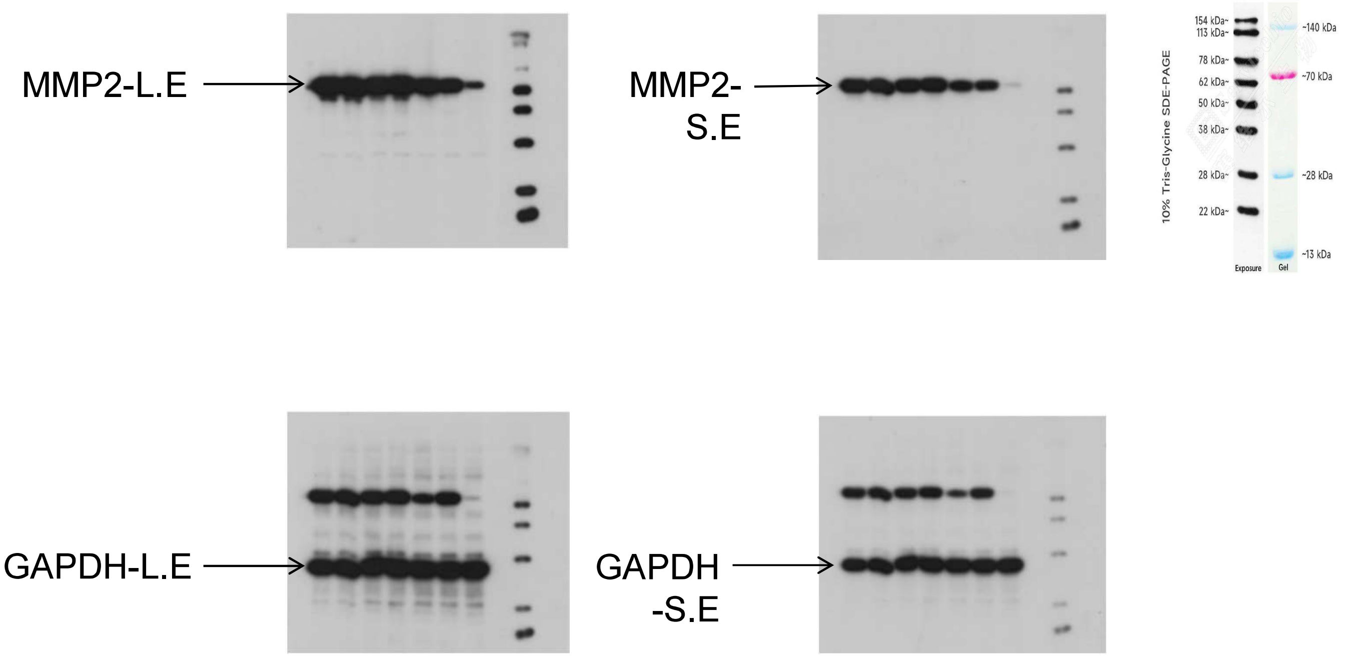

Raw Western blots

MMP9-L.E

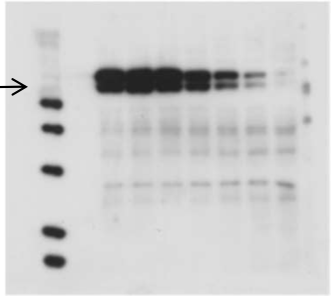

MMP9-S.E

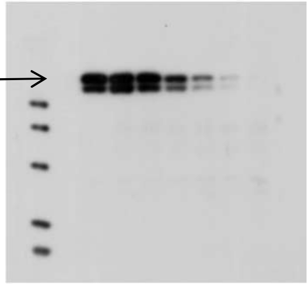

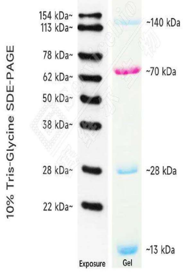

GAPDH-L.E

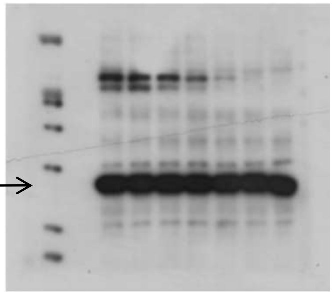

GAPDH
-S.E

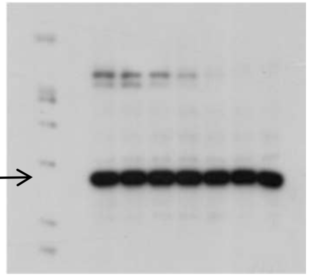

Raw Western blots

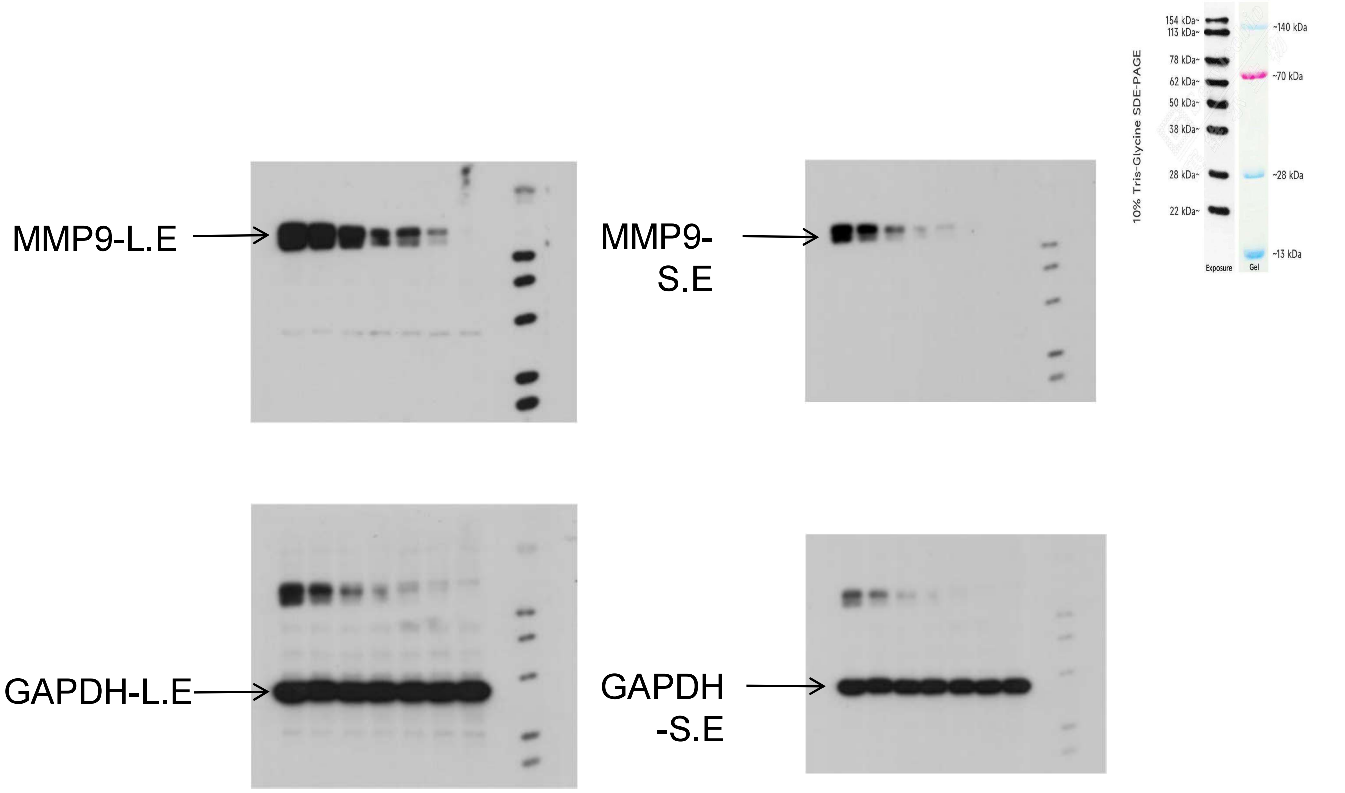

Raw Western blots

CITDE2-L.E

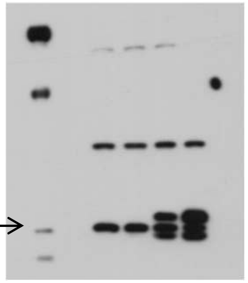

CITDE2
-S.E

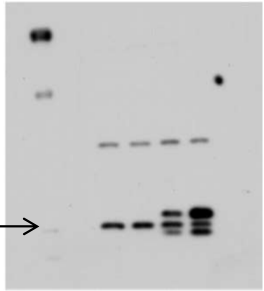

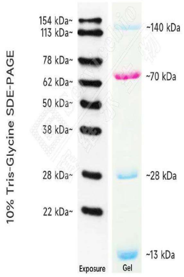

GAPDH-L.E

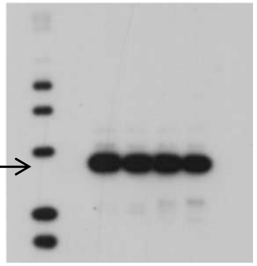

GAPDH
-S.E

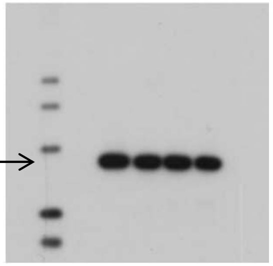

Raw Western blots

CITDE2-L.E

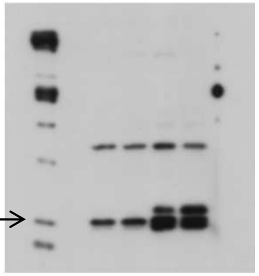

CITDE2
-S.E

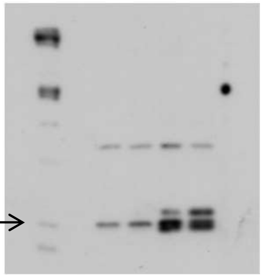

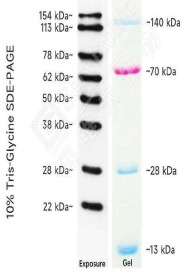

GAPDH-L.E

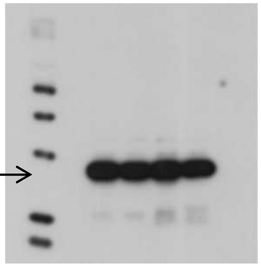

GAPDH
-S.E

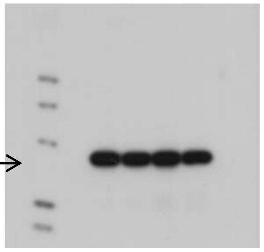

Raw Western blots

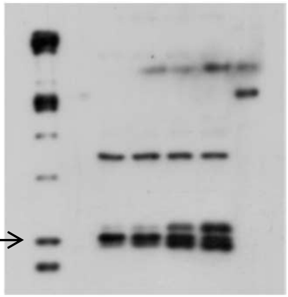

CITDE2-L.E

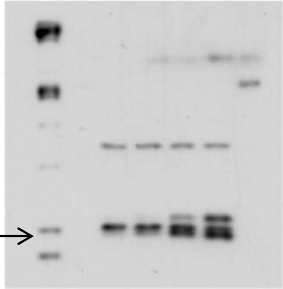

CITDE2-S.E

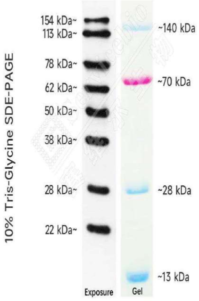

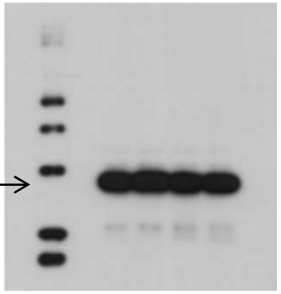

GAPDH-L.E

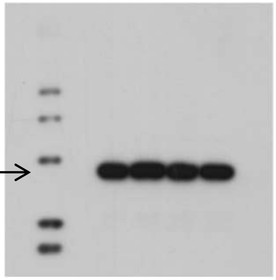

GAPDH-S.E

Raw Western blotsv

SF1-L.E

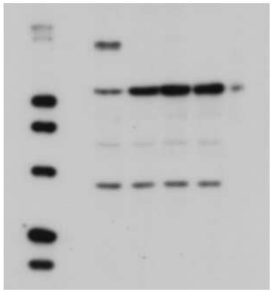

SF1-S.E

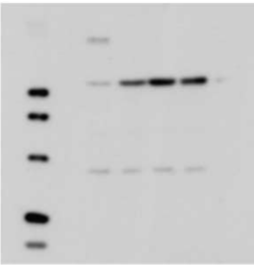

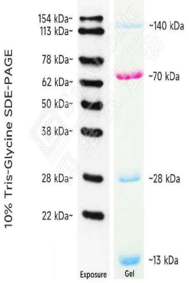

GAPDH-L.E

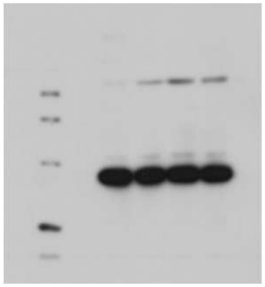

GAPDH-S.E

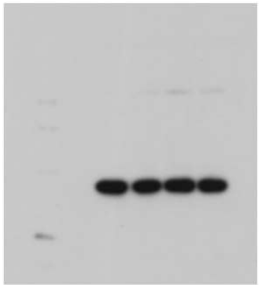

Raw Western blots

SF1-L.E

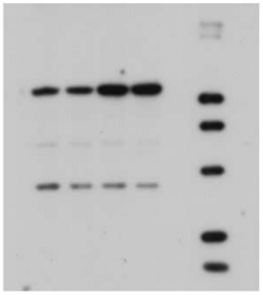

SF1-S.E

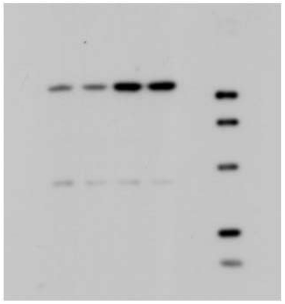

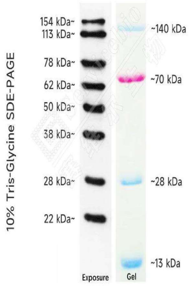

GAPDH-L.E

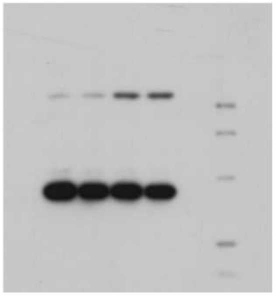

GAPDH-S.E

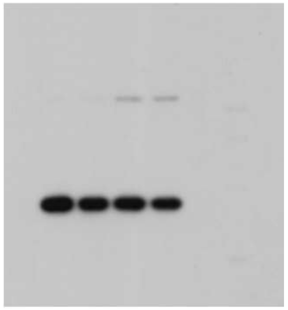

Raw Western blots

SF1-L.E

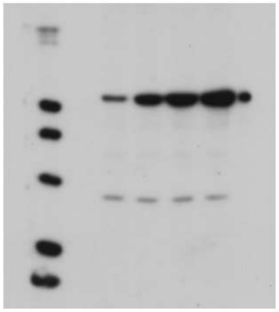

SF1-S.E

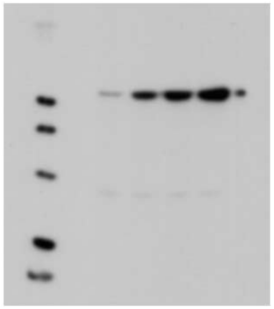

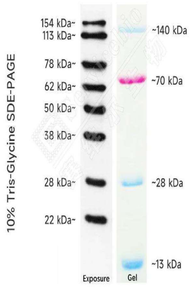

GAPDH-L.E

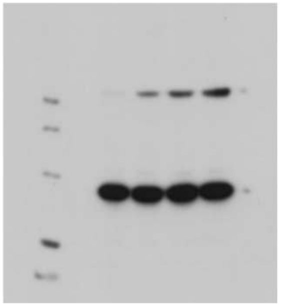

GAPDH-S.E

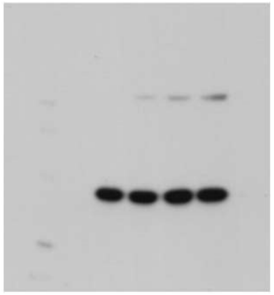

Raw Western blots

AR-L.E

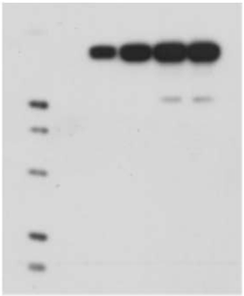

AR-S.E

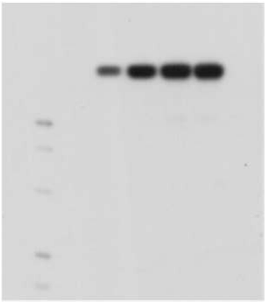

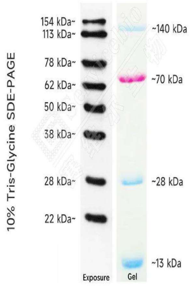

GAPDH-L.E

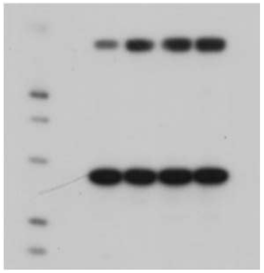

GAPDH-S.E

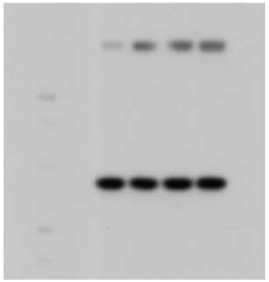

Raw Western blots

AR-L.E

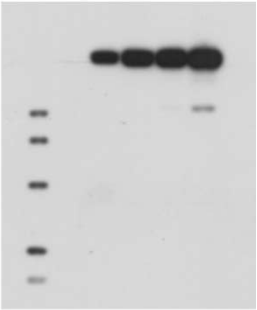

AR-S.E

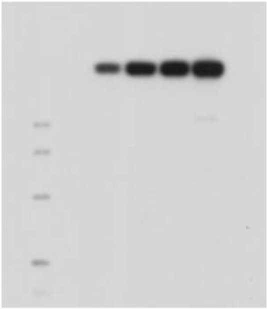

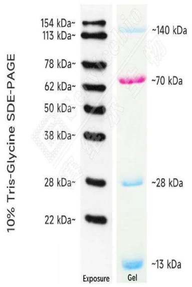

GAPDH-L.E

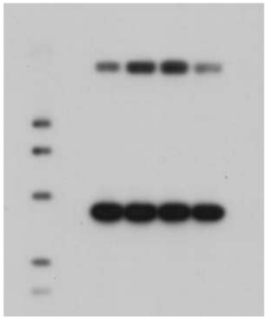

GAPDH-S.E

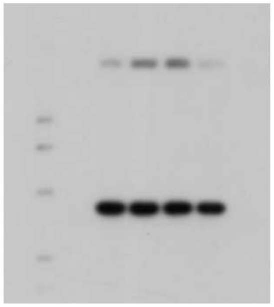

Raw Western blots

AR-L.E

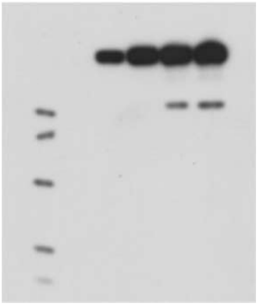

AR-S.E

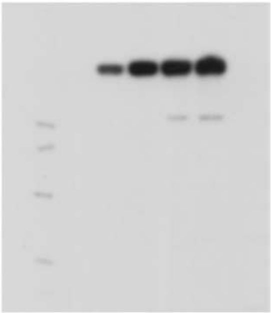

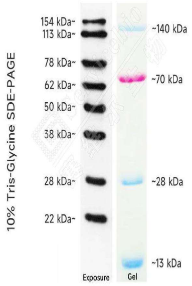

GAPDH-L.E

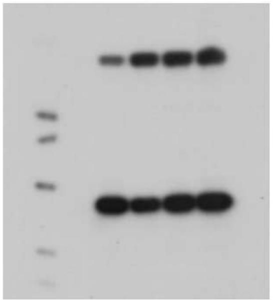

GAPDH-S.E

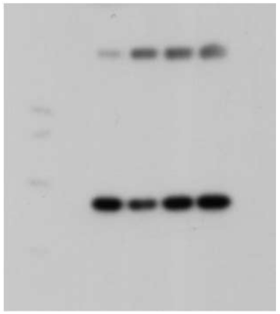

Supplement: Supplementary file 8 — Supplementary Material 8 [file 41065_2025_581_MOESM8_ESM.pdf]
